# Trait-customized sampling of core collections from a winter wheat genebank collection supports association studies

**DOI:** 10.3389/fpls.2024.1451749

**Published:** 2024-10-02

**Authors:** Marcel O. Berkner, Yong Jiang, Jochen C. Reif, Albert W. Schulthess

**Affiliations:** Breeding Research Department, Leibniz Institute of Plant Genetics and Crop Plant Research (IPK) Gatersleben, Seeland, Germany

**Keywords:** core collections, genebank genomics, association study, plant genetic resources, wheat

## Abstract

Subsampling a reduced number of accessions from *ex situ* genebank collections, known as core collections, is a widely applied method for the investigation of stored genetic diversity and for an exploitation by breeding and research. Optimizing core collections for genome-wide association studies could potentially maximize opportunities to discover relevant and rare variation. In the present study, eight strategies to sample core collections were implemented separately for two traits, namely susceptibility to yellow rust and stem lodging, on about 6,300 accessions of winter wheat (*Triticum aestivum* L.). Each strategy maximized different parameters or emphasized another aspect of the collection; the strategies relied on genomic data, phenotypic data or a combination thereof. The resulting trait-customized core collections of eight different sizes, covering the range between 100 and 800 accession samples, were analyzed based on characteristics such as population stratification, number of duplicate genotypes and genetic diversity. Furthermore, the statistical power for an association study was investigated as a key criterion for comparisons. While sampling extreme phenotypes boosts the power especially for smaller core collections of up to 500 accession samples, maximization of genetic diversity within the core collection minimizes population stratification and avoids the accumulation of less informative duplicate genotypes when increasing the size of a core collection. Advantages and limitations of different strategies to create trait-customized core collections are discussed for different scenarios of the availability of resources and data.

## 1 Introduction

About 7.4 million accessions of crop varieties, landraces, and crop-wild relative species are preserved in more than 1,750 *ex-situ* genebanks worldwide (FAO, 2010). Cereal crops dominate genebank collections (Ramirez-Villegas et al., 2022), with wheat (*Triticum* spp.), rice (*Oryza* spp.), and barley (*Hordeum* spp.) being on the leading positions with more than 850 thousand, 770 thousand, and 460 thousand accessions, respectively (FAO, 2010). These accessions are suspected to harbor beneficial alleles which are associated with an agronomic trait and got accidentally vanished from breeding germplasm due to selective bottlenecks (Tanksley and McCouch, 1997). For instance, 76% and 71% of the total variation in landraces of barley and respectively, wheat is already preserved in genebank collection (Ramirez-Villegas et al., 2022). The identification of donor genotypes retaining these beneficial alleles is vital to exploit the potential of genebanks for breeding. Nevertheless, this task remains difficult even though the results of phenotypic screenings and cost-effective high throughput genotyping are becoming progressively available for many collections. Genome-wide association studies (GWAS) benefit from, or even require, high-quality phenotyping and genotyping. At the scale of an entire collection, the all-encompassing evaluation of every single accession remains however unrealistic in terms of resources required and the sheer number of accessions. This aspect is especially valid for traits demanding expensive phenotyping protocols or for low-heritable traits as well as high-density genotyping such as whole-genome-sequencing. Consequently, more precise examination should be restricted to a targeted selection of accessions: a core-collection (CC).

Since the concept of CC was introduced (Frankel, 1984), CC have been generated based on many genebank collection for crops such as barley (Muñoz-Amatriaín et al., 2014; Milner et al., 2019), groundnut (*Arachis hypogaea* L.) (Upadhyaya et al., 2003), pepper (*Capsicum* spp.) (Lee et al., 2016), potato (*Solanum tuberosum* L.) (Esnault et al., 2016), soybean (*Glycine max* (L.) Merr.) (Bandillo et al., 2015; Haupt and Schmid, 2020), and wheat (Pascual et al., 2020; Phogat et al., 2021). Analyzing the literature spanning a recent decade, Gu and collaborators (Gu et al., 2023) discovered CC for 146 species of crops, ornamentals and trees. In general, there are two contrasting aims for selecting a CC: to represent the diversity harbored in the total collections or to maximize the diversity represented (Marita et al., 2000). Regardless of the aim, sampling strategies of CC have relied on various types of pre-existing or newly generated data such as genotypic (Esnault et al., 2016; Milner et al., 2019; Haupt and Schmid, 2020; Guo et al., 2022) or phenotypic data (Upadhyaya et al., 2003; Phogat et al., 2021); occasionally, involving a stratification based in passport data (Upadhyaya et al., 2003). Moreover, sampling of a CC may face restrictions due to the attributes of the genebank collection, the available resources, and the nature of the crop species *per se*. Certainly, there is not a uniform and universal sampling strategy for a CC (Gu et al., 2023).

Even though many CCs have been analyzed with modern biometric approaches such as GWAS (Muñoz-Amatriaín et al., 2014; Bandillo et al., 2015; Milner et al., 2019), hardly any CC has been reported to be specifically optimized for this approach. Therefore, sampling strategies for CC needs to be re-evaluated and possibly redefined in order to increase the statistical power for the identification of marker-trait-associations that are meaningful for breeding. Based on theoretical knowledge, such a CC should display specific characteristics which are summarized below.

First, the selection of accessions should be enriched with distinct alleles associated with the trait of interest and not yet present in the breeding germplasm. Extremely rare alleles with moderate effect-sizes will probably remain unnoticed in an association study; however, common variants are most likely already present in breeding programs and therefore, not of interest for the identification of donor genotypes. Generally, this also implies that distinct sets of accessions would be sampled for every trait since genetic architectures are in the best case only partially shared and mostly mismatched between different non-correlated traits. To distinguish from the classical CC, this study will refer to a trait-customized core collection (TCCC).

Second, the characteristics of such a TCCC should enable high statistical power in GWAS. The statistical power is defined as the probability to prevent a type 2 error; meaning the probability to correctly identify a marker’s association with the trait. The estimates of statistical errors depend on the probability distributions of the null hypothesis and the alternative hypothesis as well as the chosen level of significance. While choosing a more relaxed significance level will increase the power of GWAS, the specificity of a GWAS will decline. Therefore, changing the probability distributions by the constitution of the examined panel remains the only adequate way of adjusting the statistical power. Using a Q + K model, Wang and Xu (2019) demonstrated how the power can be deduced from the non-centrality parameter of the *χ²*-distribution. Proven based on simulated data, the determinants of the power are the number of genotypes in the sample, the polygenic contribution to the phenotypic variance, and the effect size of the targeted quantitative trait locus (Wang and Xu, 2019). In contrast, the present work aimed at a practical-oriented evaluation based on real datasets. Sampling extreme phenotypes, also known as selective genotyping (Van Gestel et al., 2000; Xing and Xing, 2009), is known to leverage the statistical power in GWAS (Xing and Xing, 2009; Guey et al., 2011) and was therefore implemented in five out of eight sampling strategies for TCCCs tested in the present report.

Third, population structure and genetic relationship confounded with the variation in the targeted trait can strongly interfere with GWAS results (Myles et al., 2009). Related genotypes have many genetic variants in common; vice versa, genotypes from different subpopulations share very few variants. While all of these shared or unshared variants explain the relatedness, only few variants are truly associated with the trait of interest. Therefore, a higher number of false positive associations will be discovered if population structure and genetic relationships are ignored. Myles and collaborators (Myles et al., 2009) concluded that the covariance of phenotypic similarity and relatedness describes this impact on association mapping and therefore, the correlations between phenotypic and genotypic distance can arguably be considered as a measure of population stratification. The selection of a TCCC should limit the impact of population stratification, for example by selecting accessions from the entire diversity space of the collections. Active selection against stratification is, however, complex and may negatively influence other aims such as a high number of novel beneficial variants in the TCCC.

Increasing the number of accessions in the TCCC could mitigate the negative impact of population stratification; simultaneously, the power of GWAS will as a tendency increase with the number of accessions (Wang and Xu, 2019). Moreover, the number of distinct positively associated alleles in the TCCC should approach a collection-specific maximum as the number of accessions in the TCCC increases. In general, an unlimited increase in the number of accessions would therefore be an easy, though unrealistically expensive approach to address all problems identified above. Not only the costs of high-quality phenotyping and genotyping, but also the cost for maintaining a TCCC in pure constant conditions on the long-term, urge the need to limit the size of a TCCC to the effective minimum. In addition, a larger TCCC will increase the chance of sampling accessions which are genetic duplicates. Some genebank collections have been reported to harbor duplicate accessions with for instance an abundance of 37% (Schulthess et al., 2022a) and even up to 54% (Singh et al., 2019), respectively. Undoubtedly, the size of a TCCC has therefore an extreme impact. Sizes of CC have mostly been defined relatively to the size of the original genebank collection. Brown (1989a) recommended a proportion of 10% of all available accessions and found to cover about 70% of the entire genetic variations with this CC size. Depending on the collection, these proportions can in practice vary a lot (Van Hintum et al., 2000) and can be translated into a few or more than thousand accessions (Gu et al., 2023). We advance the hypothesis that the composition of the TCCC, as defined by the underlying sampling strategy, determines the success of the identification of marker-trait-associations and the corresponding donor genotypes, given a constant size of the TCCC.

The main goal of the present study was to compare the effect of different sampling strategies on the probability to identify donor genotypes for two agronomic traits in the winter wheat collections of the German Federal *Ex situ* Genebank hosted at the Leibniz Institute of Plant Genetics and Crop Plant Research (IPK Genebank). In particular, the objectives were to (1) elaborate eight different selection strategies, relying on phenotypic and/or genotypic information, to sample accessions for TCCCs of increasing size for two traits, namely yellow rust susceptibility (YR) and stem lodging (SL), (2) study the suitability of the strategies to identify association in GWAS based on power estimates of trait-specific panels of markers, genetic and phenotypic diversity in the TCCC as well as population stratification, and (3) investigate the impact of the size of the TCCC on the power of quantitative trait locus detection.

## 2 Materials and methods

### 2.1 Plant material

The presented research relied on a previously published subset of the *Triticum* collection stored at the IPK Genebank (Schulthess et al., 2022a, b). In total, 7,651 accessions were selected with the intention to include most of the available winter wheat accessions. While the vast majority were *Triticum aestivum* L., nine accessions were classified as other species of the *Triticum* genus.

### 2.2 Genotypic data

Genotyping-by-sequencing profiles for all 7,651 accessions were used as previously published by Schulthess and collaborators (Schulthess et al., 2022a, b). Concisely, genotypic profiles were generated by engaging a genotyping-by-sequencing approach as follows. On the field, 7,745 distinct morphotypes were identified among all 7,651 genebank accessions. Each morphotype was transferred into an isolate line by bagging one characteristic ear and propagation in an ear-to-row fashion; the isolate line is referred to as accession sample. For each isolate line, DNA was extracted from a 10-day-old seedling and subsequently digested using PstI and MspI restriction enzymes followed by ligation to adapters comprising barcode sequences. Digested samples were pooled into genotyping-by-sequencing libraries and sequencing was performed either on an Illumina Hiseq-2500 or a NovaSeq 6000 system. The resulting reads were trimmed to a minimum read length of 30 bp followed by SNP calling based on the wheat reference genome var. Chinese Spring v. 1.0 (IWGSC, 2018). The resulting SNP matrix was further filtered: only markers with less than 10% missing values, any of both alleles in homozygous state in at least 10 genotypes, and heterozygosity ≤1% were retained for downstream analyses. Finally, missing genotypic data was imputed marker-wise as the predominant allele among all accession samples.

### 2.3 Phenotypic records and performance estimation

Two phenotypic traits were considered in the present work: susceptibility to yellow rust infections (YR) and stem lodging (SL). Both traits were measured by following the standard protocols of the German Federal Plant Variety Office (Bundessortenamt, 2020) and based on 1-9 scoring scales, with 1 corresponding to YR resistance or SL tolerance, and 9 indicating extreme YR or SL susceptibility. YR and SL were recorded in 12 and 13 field experiments, respectively, with ten experiments having records for both traits simultaneously. YR was based on natural infections and recorded when sufficient disease pressure was observed for an entire experiment while SL was measured after heading stage. Field experiments involved two German locations, Gatersleben (latitude 51° 49’ 19.74’’ N, longitude 11° 17’ 11.80’’ E) and Schackstedt (latitude 51° 43’ 0’’ N, longitude 11° 37’ 0’’ E), as well as seven consecutive growing seasons within the 2014-2020 period. Each experiment was performed to large-scale test 1,428-1,698 entries under an alpha-lattice design and considering a 0.4 m² plot as experimental unit. All experiments were performed considering two replications, but in two experiments phenotypes were recorded in only one replication. Experiments were partially connected through overlapping common entries. Crop management of all experiments considered only the chemical control against weeds, while no fertilizers were additionally applied. Data quality assessment as well as the computation of variance components for heritability estimation and the average performance across experiments, best linear unbiased estimates (BLUEs) for each trait and entry were performed based on linear mixed models as implemented in the R software package ASReml-R (v. 3) (Butler et al., 2009). Further details on methods underlying these analyses can be found in past works (Schulthess et al., 2022a, b), where YR phenotypic records were originally published. SL data has not been either presented or assessed previously. Out of the 7,745 accession samples with genotyping-by-sequencing profiles, 6,300 and 6,251 have BLUEs for YR and SL, respectively, and were used for downstream analyses.

### 2.4 Evaluation of genetic diversity and population structure

Genetic diversity within groups of accessions was analyzed based on genetic distances. With this intention, pairwise Rogers’ distances were calculated (Rogers, 1972) for all 7,745 accession samples and condensed by principal coordinate (PCo) analysis (Gower, 1966). Population structure was visualized by plotting first and second PCos against each other.

### 2.5 Tested sampling strategies

Eight different strategies to sample a TCCC were contrasted in the present study. The tested strategies differed with respect to the data sources required for the selection decision as outlined in **Table 1**. While four sampling strategies relied on phenotypic information, one strategy required exclusively genomic profiles and additional two strategies engaged both types of data. For the latter, phenotypic data, as BLUEs per accession, were linked to the existent genotypic data through accession samples. Only one strategy did not need any data: completely random sampling (All_random) of accessions was included as benchmark for the most simplistic approach. In the second sampling strategy (All_Pdiv), the range of phenotypic values of all accession samples was subdivided into 10 equally-large quantiles, while the total sample size was subdivided into individual drawings which were randomly assigned to the 10 defined quantiles. During implementation, restrictions due to fewer representatives toward the extremes of distributions must be considered. Therefore, if the number of drawings was larger than the total number of representatives within the respective quantile, the surplus was randomly distributed to the other quantiles. Thereafter, accession samples were randomly sampled within the quantiles according to the number of drawings. In this way, the whole range of phenotypic diversity was covered as equally as possible. For the third sampling strategy (All_Gdiv), the R software package corehunter (v. 3.2.1) (De Beukelaer and Davenport, 2018) was used to maximize the genetic diversity within the TCCC. The algorithm, as implemented in the “sampleCore” function, was applied with the specification EN (entry-to-nearest-entry distance) in combination with MR (modified Roger’s distance) and allowing a maximum of 20 iterations without any improvement. This function run in combination with rJava (v. 1.0-6) (Urbanek, 2021).

**Table 1:** Description of the eight strategies applied for sampling of trait-customized core collections.

Name	Description	Phenotypic data	Genotypic data
All_random	Completely random sampling from all accession samples	No	No
All_Pdiv	Sampling phenotypes equally covering the entire range of the phenotypic diversity	Yes	No
All_Gdiv	Sampling from all accession samples in order to maximize genetic diversity	No	Yes
1T_rank	Sampling from the tail of best-performing accession based on phenotypic ranking	Yes	No
2T_rank	Sampling the most contrasting phenotypes from two tails based on phenotypic ranking	Yes	No
2T_random	Random sampling of accession samples from two phenotypic tails	Yes	No
2T_Gdiv&Gsim	Maximizing genetic diversity within the tail of best-performing accession and sampling of related genotypes from the contrasting tail	Yes	Yes
2T_Gdiv&Gdiv	Maximizing genetic diversity independently within both contrasting phenotypic tails	Yes	Yes

Depicted are the abbreviated names of the strategies, a short description of the underlying procedure as well as an indication about the required data.

In the fourth sampling strategy (1T_rank), all accessions were ranked based on the phenotypic values and the most resistant accessions, thus with the lowest BLUEs, were successively selected. Similarly, the sampling strategy (2T_rank) involved a ranking of accessions based on their phenotypic values. However, accessions were chosen from the lowest and highest phenotypic range in a 3:1 ratio. For both traits, low phenotypic scores are the breeding target and thus, this last step allows the accumulation of beneficial variants within the TCCC.

For the remaining three strategies, a positive and a negative tail were defined based on the distribution of BLUEs. Each phenotypic tail contained the 10% of all accession which have the lowest and highest phenotypic values. Tails with low and high values are referred to as positive and negative tail, respectively. Random sampling in the positive and negative tail was performed for the strategy called 2T_random, while the tails were considered in a 3:1 ratio. For the last two tested sampling strategies, the R software package corehunter (v. 3.2.1) (De Beukelaer and Davenport, 2018) was engaged to maximize the genetic diversity among the accessions chosen from the positive tail. For this maximization, the same software specifications were used as described above for All_Gdiv. For the seventh sampling strategy (2T_Gdiv&Gsim), accessions were sampled from the negative tail in a 3:1 ratio in such a way that one accession from the negative tail was chosen due to the lowest Rogers’ distances with three accessions of the positive tail. This stepwise selection was repeated until all accessions from the positive tail were covered. For the eighth sampling strategy (2T_Gdiv&Gdiv), accessions were initially sampled from the negative tail based on a low genetic distance in exactly the same number as accessions were present in the positive tail. In a second step, the number of accessions in the pre-selection from the negative tail was reduced in order to achieve the 3:1 ratio between the samples from the positive and negative tail. For the latter step, the R software package corehunter (v. 3.2.1) (De Beukelaer and Davenport, 2018) was used as described above to sample a lower number of accessions in parallel to maximizing the genetic diversity within the sample from the negative tail.

The described sampling strategies were implemented for eight different sizes of TCCCs, measured in the number of accession samples included. The sizes of TCCCs increased with a step size of 100. The minimum size was set at 100 because this is the widely-accepted threshold in GWAS to yield publishable results; in contrast the maximum of 800 reflects the size of the phenotypic tails and the 3:1 sampling ratio between both tails in some sampling strategies. The sampling and later evaluation was performed with 50 independent replications for each combination of strategy and size of TCCC.

### 2.6 Evaluation of trait-customized core collections

The characteristics of the TCCCs differed depending on the compositions of selected accession samples. Unbiased comparisons of TCCCs and the associated sampling strategies were ensured by only contrasting TCCCs of the same size. The comparison was based on six criteria which were namely the phenotypic distribution, the correlation between phenotypic and genotypic distances, the number of duplicate genotypes based on identity-by-state values between sampled accessions, the genetic distinctness of the sampled TCCC from the remaining accessions samples (F_st_), the statistical power of GWAS (Wang and Xu, 2019), and the average minor-allele-frequency (MAF) within the TCCC and within the trait-specific panels of markers which are later on referred to as Top10_MTAs.

For each trait, the Euclidean distances were computed separately for all pairs of accession samples in the TCCC or in the entire collection and the correlation between these values and the pairwise Rogers’ distances from genomic data served as diagnostic measure for population stratification. Identity-by-state values were calculated for all pairwise comparisons within the respective TCCC with the “snpgdsIBS” function of the R software package SNPRelate (v. 1.24.0) (Zheng et al., 2012). A threshold of identity-by-state values > 99% was applied to declare two accession samples as duplicates, as similarly done by Schulthess and collaborators (2022a). The pairwise F_st_ was calculated between all accession samples in the TCCC and all remaining accession samples of the genebank collection. The pairwise F_st_ was calculated with the “fs.dosage” function of the R software package hierfstat (v. 0.5-11) (Goudet and Jombart, 2022). The effective population size ( $N_{e}$ ) was calculated within each TCCC based on (Waples, 2006):


(1) $${\text{N}}_{\text{e}}=\frac{1}{3\left({\text{r}}_{\text{linkage}}^{2}-\frac{1}{\text{n}}\right)}\;$$

in which *n* is the number of sampled genotypes and $r_{linkage}^{2}$ denotes the mean linkage disequilibrium of unlinked markers in the sampled genotypes. In order to ensure the unlinked state, linkage disequilibrium was calculated as the average squared correlation between markers located on different chromosomes.

The statistical power of GWAS was estimated following the theoretical approach of Wang and Xu (2019). More precisely, we first performed GWAS in the total set of accession samples and took the 10 most significant markers as hypothetical QTL for each trait, referred as trait-specific Top10_MTAs. The computations for GWAS relied on the “GWAS” function of the R software package rrBLUP (v.4.6.1) (Endelman, 2011); the minimum MAF was set to 1%. Then, the approach of Wang and Xu (2019) was used to estimate the power of detecting these markers in the TCCCs. For a GWAS study relying on a Q+K model and involving *n* genotypes, the power of detecting a specific marker can be estimated based on the non-centrality parameter, *δ*, of a *χ²*-distribution, as follows (Wang and Xu, 2019):


(2) $$\text{δ}=\left(\text{λ}+1\right)\sum_{\text{j}=1}^{\text{n}}\frac{1}{\left({\text{d}}_{\text{j}}\text{λ}+1\right)}\frac{r_{\text{marker}}^{2}}{1-r_{\text{marker}}^{2}}$$

in which $d_{j}$ are the eigenvalues of the kinship matrix, $r_{marker}^{2}$ is the proportion of phenotypic variance explained by the marker estimated within the TCCC and λ represents the ratio of polygenic variance to the residual variance. The genetic variance and residual variance were calculated with the “kin.blup” function of the R software package rrBLUP (v.4.6.1) (Endelman, 2011) and considering all accession samples within the TCCC with BLUEs for the trait of interest. A significance level of 0.05 was applied in combination with the simpleM method (Gao et al., 2008) to account for the bias due to multiple testing. The R software package ggplot2 (v. 3.4.4) (Wickham, 2016) was used for the visualization of the results. Manhattan plots were created with the R software package qqman (v. 0.1.4) (Turner, 2018). All computations were performed in the R environment (v. 3.4.4, v. 4.0.2) (R Core Team, 2020).

## 3 Results

### 3.1 Phenotypic distributions and trait’s association with population structure

**Figure 1:**
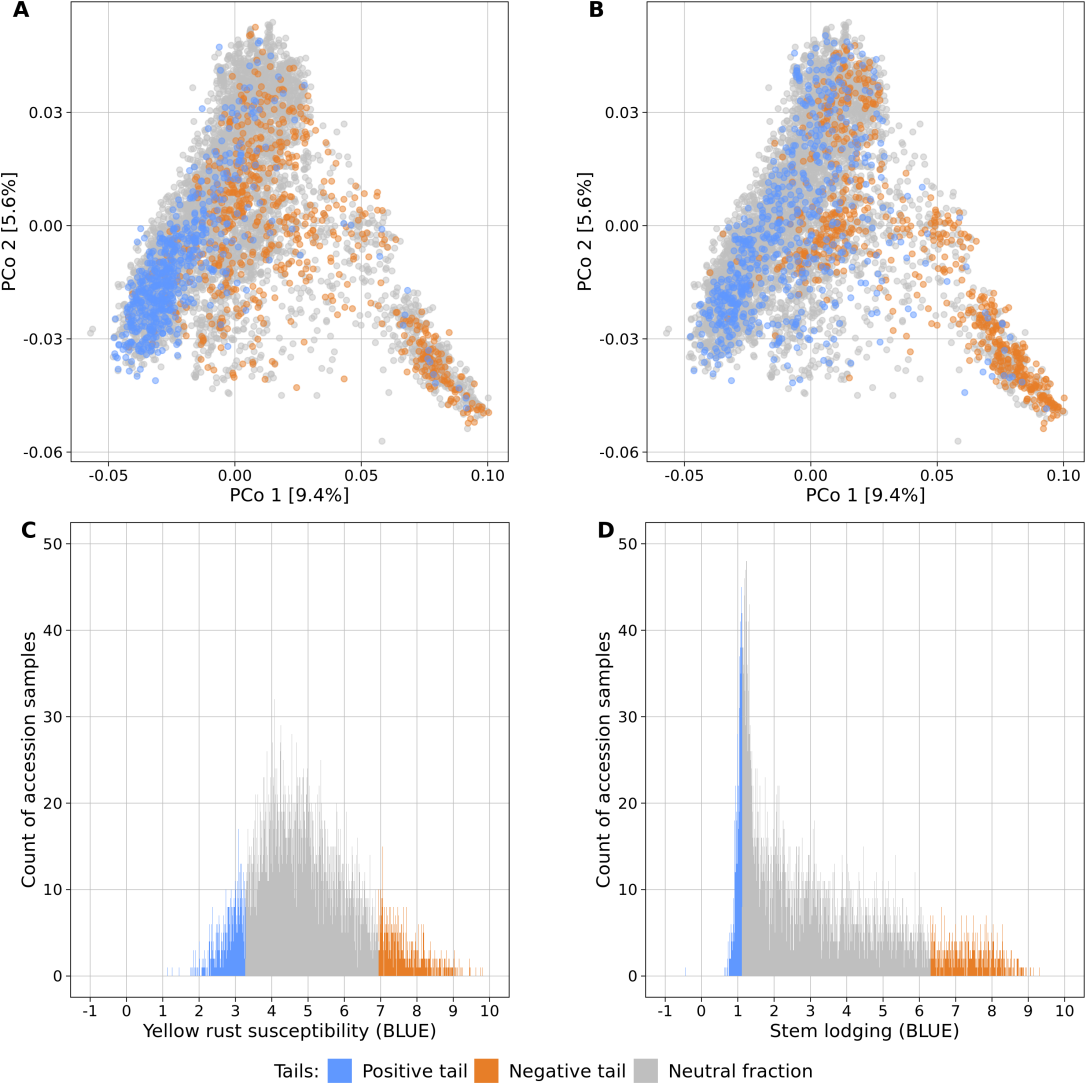
Genetic diversity and phenotypic distributions of the two contrasting phenotypic tails each containing 10% of the accessions samples with the lowest (blue) and highest (orange) phenotypic values of yellow rust susceptibility **(A, C)** and stem lodging **(B, D)**. The remaining 80% of all accession samples are shown in gray color. The phenotypic distributions are plotted in relation to the genetic diversity **(A, B)** which is displayed as a biplot of the first and second principal coordinates (PCo) from the Rogers’ distances between 7,745 accession samples of the IPK winter wheat collection. Histograms display the abundance of best linear unbiased estimates (BLUE) for yellow rust susceptibility **(C)** and stem lodging **(D)** along the phenotypic range. The phenotypic values exceed the 1-9 range of the phenotyping scale due to the non-orthogonal structure of characterization experiments across locations and years and the computation of linear mixed models.

Fifteen large-scale field experiments provided data with very high heritabilities for YR (*h²* = 0.82) and SL (*h²* = 0.86) (**Supplementary Table S1**) as well as BLUEs for 6,300 and 6,251 accession samples for YR and SL, respectively. The distributions of the traits were distinct: while BLUEs of YR followed a more symmetric distribution (mean = 4.95, median = 4.79, standard deviation = 1.40), SL had an L-shaped distribution which was strongly skewed towards lower values (mean = 3.18, median = 2.61, standard deviation = 2.03) (**Figure 1**).

Population stratification can strongly bias GWAS results and thus, the association of the phenotypic distributions with the population structure was investigated for both traits in the entire genebank collection. Strong correlations with the first PCo from genomic distances were observed for both YR (*r* = 0.42) and SL (*r* = 0.52), thus indicating a clear association of population structure with trait variation (**Table 2**). The separation along the first PCo can also be seen graphically based on the location of the phenotypic tails, one with low and another with high BLUEs, in the diversity space (**Figure 1**). The correlations with the second, third, fourth and fifth PCo were clearly lower in magnitude however still statistically significant (*p*-values < 0.001).

**Table 2:** Correlation coefficients for the relation between the five most-explanatory principal coordinates (PCo) calculated from the Rogers’ distances between 7,745 accession samples of the IPK winter wheat collection and the best linear unbiased estimates for the traits yellow rust susceptibility (YR for 6,300 accession samples) and stem lodging (SL for 6,251 accession samples).

	PCo 1	PCo 2	PCo 3	PCo 4	PCo 5
	(9.42%)	(5.63%)	(3.00%)	(2.06%)	(1.89%)
YR	0.4225^***^	0.0473^***^	-0.2555^***^	-0.1322^***^	-0.0984^***^
SL	0.5196^***^	0.0878^***^	0.0444^***^	0.3167^***^	-0.1050^***^

The percentages in brackets display the amount of molecular variance explained by the respective PCo. ***p ≤ 0.001.

### 3.2 Selection of a marker panel showing association in the entire population

Based on all available data, GWAS was performed to identify likely associated markers that were later investigated within the sampled TCCCs. For both traits, some markers were identified as having a high probability of being associated with the trait of interest (**Supplementary Figure S1**) and the 10 markers with the highest -log_10_(*p*)-value were selected for a trait-specific marker panel and referred to as Top10_MTAs. For both traits, these markers were located on several distinct chromosomes (**Supplementary Figure S2**). The markers of the Top10_MTAs panels were later categorized based on the effect size, i.e. large, medium, and small *r²* values, and the MAF (common, rare) (**Supplementary Table S2**). With a size of 10 markers, the Top10_MTAs marker panels comprised multiple markers per category; however, not all combinations of effect size and MAF were present in these panels. Common markers with a medium to large effect are often already known to breeders and incorporated in the germplasm. Four and five markers were of this type for YR and SL, respectively. Markers with a small effect demand much more effort from breeding to achieve a measurable improvement of the germplasm; three small effect markers (*r²* ≤ 0.001) were found for both traits. Markers with medium to large effects which are rather rare are arguably the primarily target of genebank genomics. For YR, markers 6A_135235117 and 2B_57683037 were classified as such while markers 2D_186808122 and 2D_186808096 were identified for SL (**Supplementary Table S2**). The latter two markers could not be distinguished for the calculation of the *r²* value due to complete collinearity.

### 3.3 Phenotypic distributions and population stratification resulting from sampling strategies

**Figure 2:**
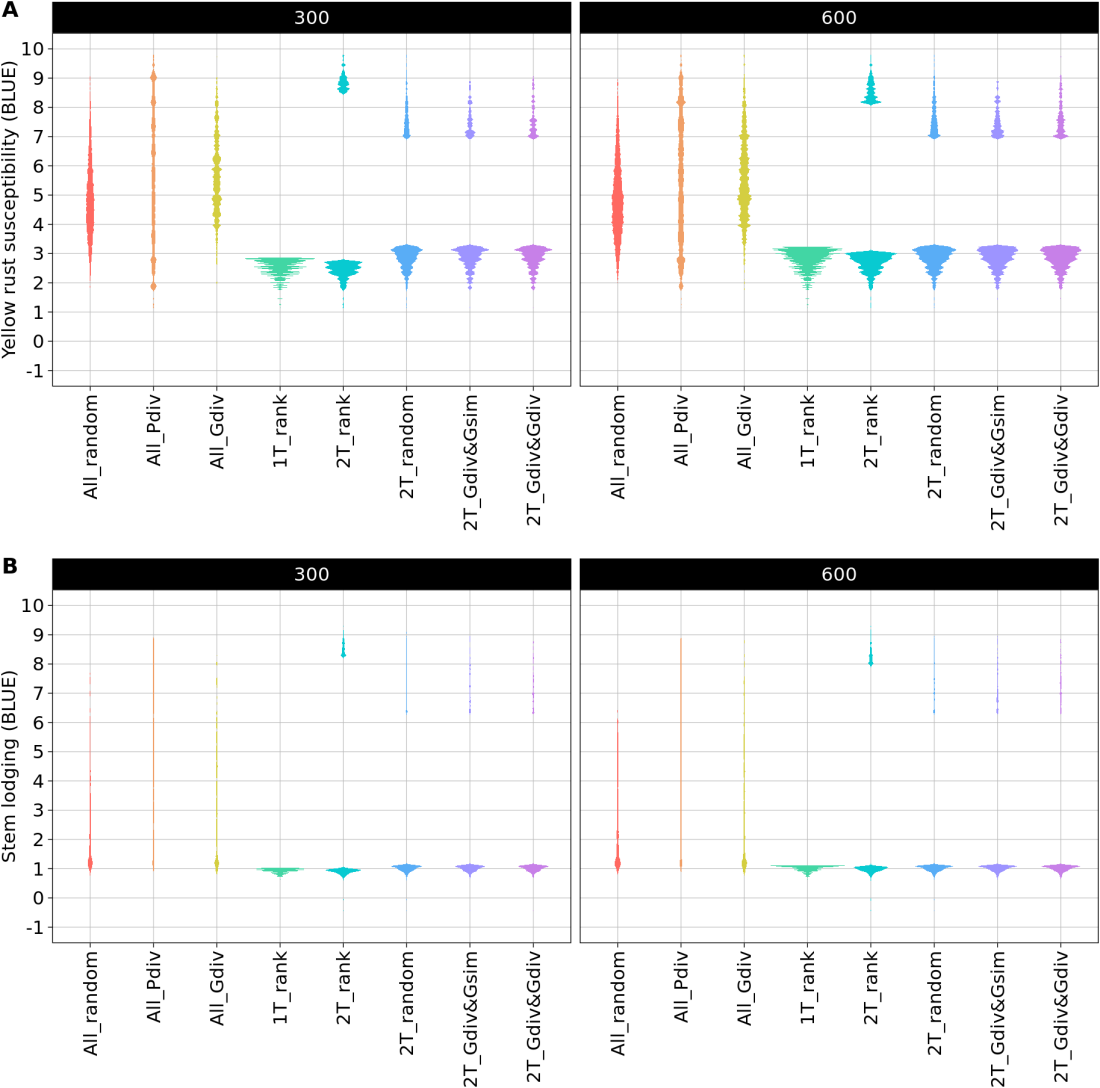
Comparison of phenotypic distributions of yellow rust susceptibility **(A)** and stem lodging **(B)** within the trait-customized core collection generated by eight different sampling strategies. Shown are the averaged distributions of best linear unbiased estimates (BLUE) seperately for 300 and 600 included accession samples. The phenotypic values exceed the 1-9 range of the phenotyping scale due to the solving of the linear mixed model.

The phenotypic distributions of the sampled TCCCs showed the expected strong differences between the sampling strategies. As an example, the distributions are presented for two TCCC sizes: 300 and 600 accession samples (**Figure 2**). The sampling strategies can be clustered into three groups: first, the random sampling (All_random) as well as the sampling along the entire phenotypic range (All_Pdiv) resulted in TCCCs that already cover the available phenotypic diversity despite of the limited number of samples in TCCCs. Similarly, All_Gdiv led to TCCCs that cover the phenotypic range of the entire collection with increasing size of the TCCC. Second, samples resulting from 1T_rank represent a small phenotypic range in combination with overall low values. Third, the phenotypic distribution associated with the strategies which relied on two contrasting phenotypic tails combine in general a broad range of phenotypic values with a high proportion of accessions with low BLUEs and entirely lack intermediate phenotypes. Differences among the latter four strategies can mostly be seen in TCCCs of smaller size since almost the entire positive tail was included for extremely large TCCCs regardless of the strategy.

**Figure 3:**
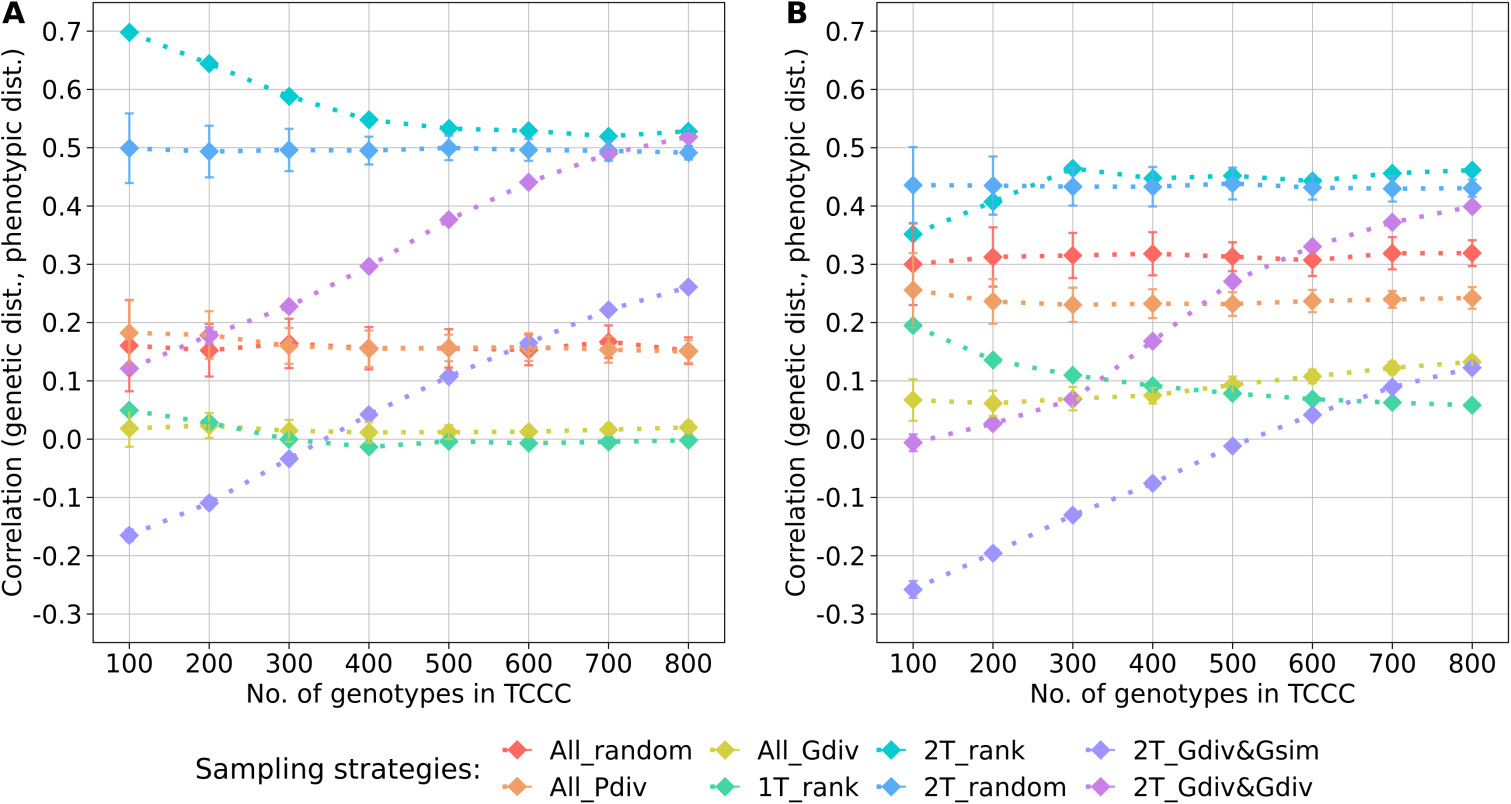
Correlation between the genetic distance (Rogers’ distance) and the phenotypic distance (Euclidean distance) depending on the number of accession samples included in trait-customized core collections for yellow rust susceptibility **(A)** and stem lodging **(B)**, respectively. Values are depicted separately for eight different sampling strategies representing mean values of 50 independent replications; whiskers display the standard deviations.

Within the sampled TCCCs, correlations between phenotypic and genotypic distances were investigated as a possible indicator of population stratification. For both traits, random sampling of accessions (All_random) resulted in a positive correlation which was fairly constant across different sizes of TCCCs. These correlations could be seen as a benchmark for the comparison with the other strategies since they represent the correlation present in the entire population (**Figure 3**). For the entire collection, the base correlations amount to 0.16 and 0.32 for YR and SL, respectively and any correlation lower than this is arguably a moderation of population stratification in the respective TCCC. For both traits, strong positive correlations were found for the two-tail sampling strategies which disregard genotypic diversity (2T_rank and 2T_random). These strategies did not only yield higher correlation values than All_random but provided also the highest correlation per trait in general: applying 2T_rank to the YR data resulted in a correlation of 0.70 for a TCCC size of 100; for SL, an overall maximum of 0.46 was found in 300 accession samples with the same strategy. Therefore, these strategies probably fortify population stratification. While 2T_Gdiv&Gsim and 2T_Gdiv&Gdiv resulted in TCCCs with low or even negative correlations for smaller TCCCs, positive correlations were found for larger TCCCs with a continuous increase in magnitude as a function of TCCC size. Minimizing phenotypic diversity including only low BLUEs (1T_rank) as well as maximizing genetic diversity regardless of the phenotype (All_Gdiv) results in TCCCs having moderate positive correlations which are lower compared with All_random.

### 3.4 Genetic diversity and representativity of the sampled TCCC

**Figure 4:**
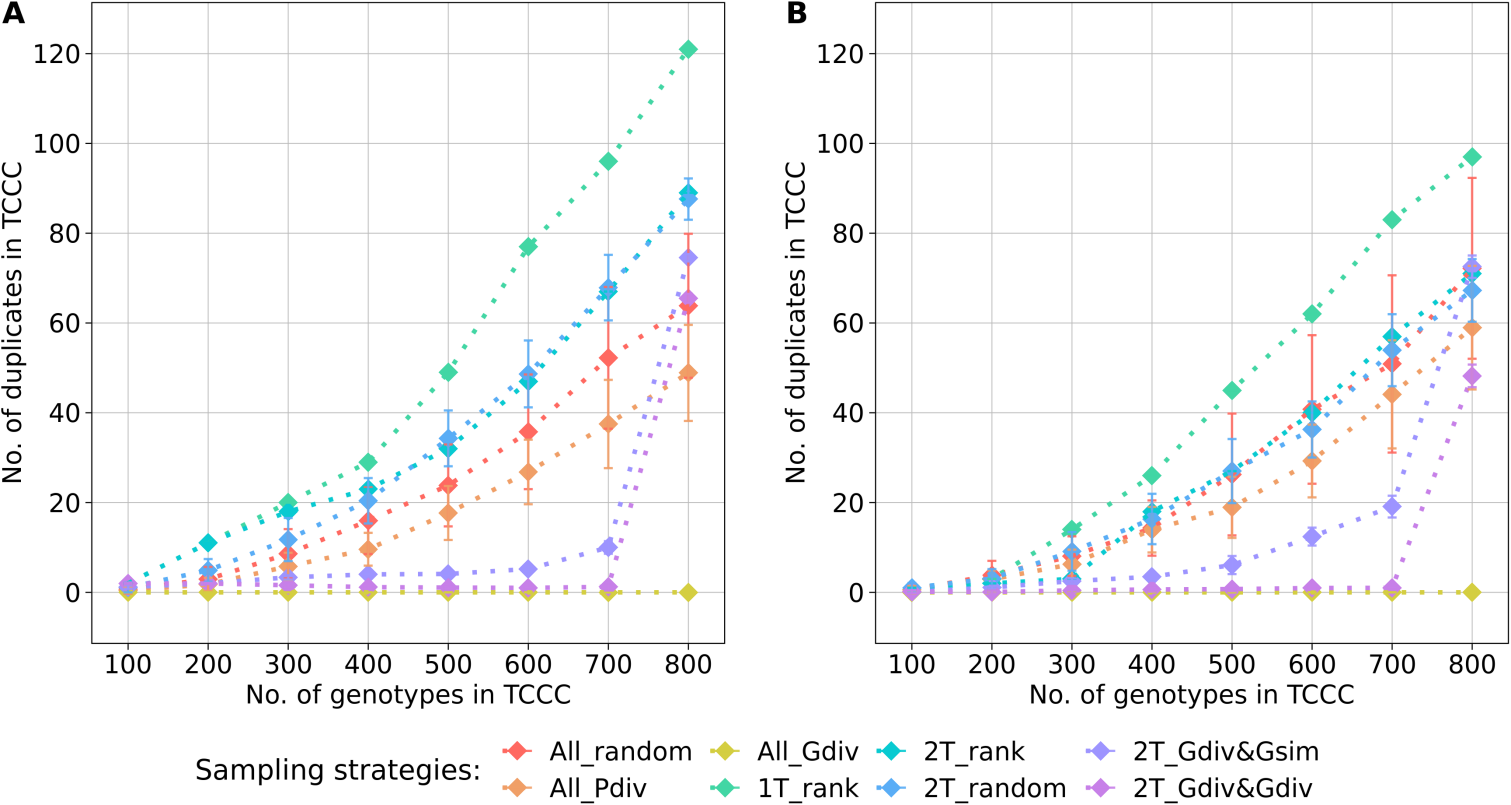
Number of duplicate genotypes, defined by an identity-by-state value higher than 0.99, depending on the number of accession samples included in trait-customized core collections (TCCC) for yellow rust susceptibility **(A)** and stem lodging **(B)**, respectively. Values are depicted separately for eight different sampling strategies representing mean values of 50 independent replications; whiskers display the standard deviations.

The genetic diversity within the TCCC and the representativity for the entire genebank collection were evaluated with three indicators that allow to rank the different sampling strategies for a fixed TCCC size. Based on the estimated effective population size ( $N_{e}$ ), the sampling strategies could be clustered into two groups (**Supplementary Tables S3**, **S4**): Sampling strategies that maximized the genetic diversity by engaging the maximizing algorithm were contrasting to strategies that do not rely on genotypic information. For all evaluated sizes of TCCCs, the All_Gdiv sampling strategy led by far to TCCCs with the highest $N_{e}$ values. When sampling TCCCs containing 300 accession samples, this sampling strategy led to maximum $N_{e}$ values of 361 and 355 for YR (**Supplementary Table S3**) and SL (**Supplementary Table S4**), respectively. For both 2T_Gdiv&Gsim and 2T_Gdiv&Gdiv, the effect of the genetic maximization on the effective population size was more dominant for SL but less pronounced for YR. Another perspective on the genetic diversity covered within TCCCs is given by the abundance of duplicate genotypes defined by the identity-by-state measure (**Figure 4**). Differences in the number of duplicates between TCCCs became more obvious for TCCCs of larger size. For YR and SL, differences could be observed starting with TCCC sizes of 200 and 300 accessions, respectively. As a general tendency, sampling strategies which rely on genotypic information sampled a lower number of duplicate genotypes which also demonstrated the opposing causal relationship with the higher $N_{e}$ values. Regardless of the trait, 1T_rank sampled by far the highest number of duplicate genotypes. A high proportion of duplicates was still sampled by applying strategies 2T_rank and 2T_random, especially for YR.

**Figure 5:**
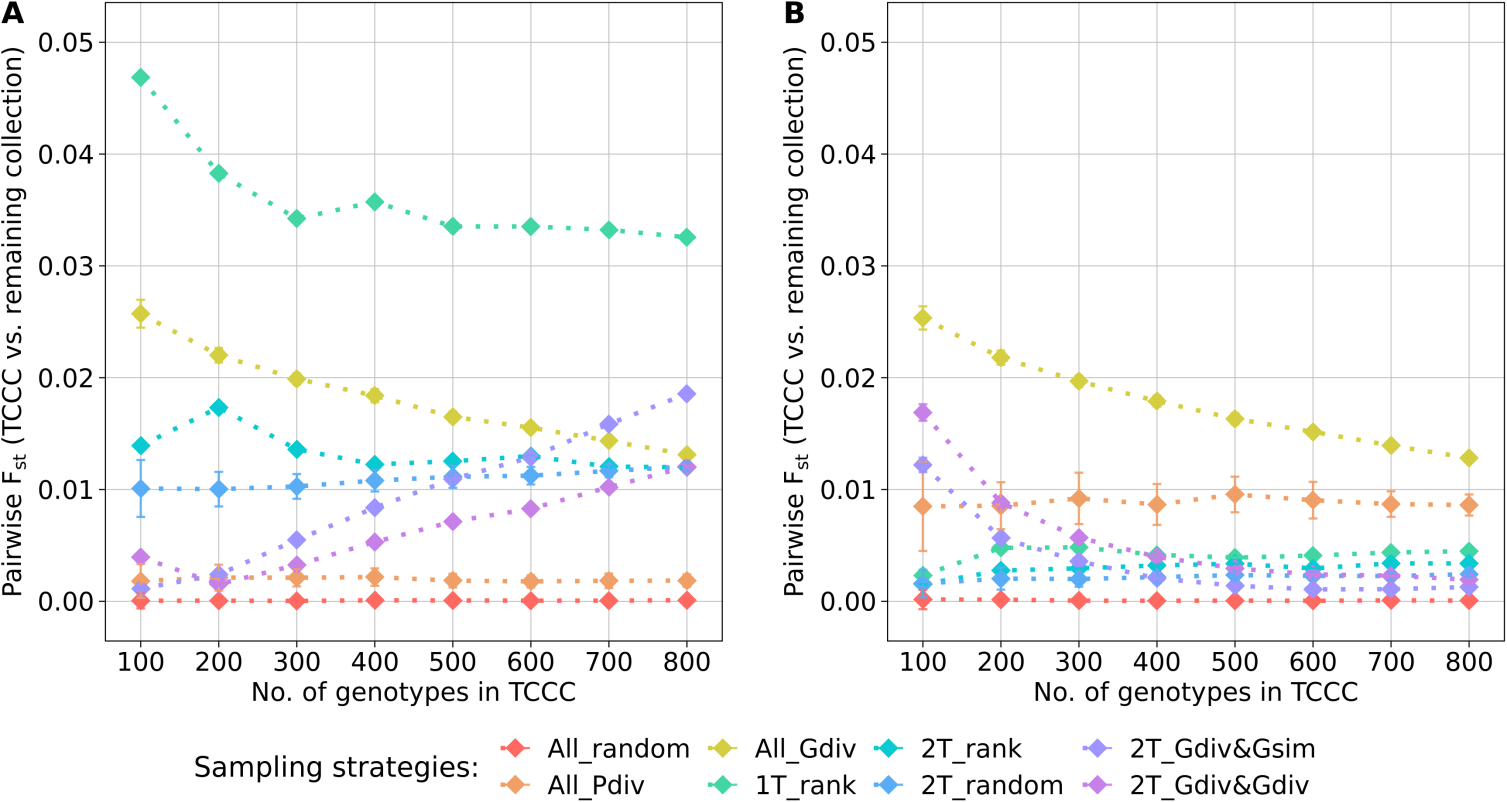
Pairwise F_st_ calculated between the trait-customized core collections (TCCC) and the remaining part of the genebank collection depending on the number of accession samples included in trait-customized core collections for yellow rust susceptibility **(A)** and stem lodging **(B)**, respectively. Values are depicted separately for eight different sampling strategies representing mean values of 50 independent replications; whiskers display the standard deviations.

The genetic representativity of the TCCCs was evaluated based on the pairwise F_st_ of the TCCC and all remaining accession samples of the entire collection (**Figure 5**). Indicated by a value in proximity to zero, random sampling (All_random) led on the one hand to a perfect genetic representation of the entire collection. On the other hand, a distinct genetic distribution in the TCCC was sampled for YR with 1T_rank and for SL with All_Gdiv, while applying 1T_rank for SL resulted in moderate values. These results not only emphasize the impact of considering the phenotypic diversity for the sampling strategy but also highlight the need to focus on trait-customized approaches.

### 3.5 Statistical power and further requirements for association studies

While the aforementioned measures describe the entire sampled TCCC, measures indicating the capability of identifying association were investigated based on the two trait-specific Top10_MTAs marker panels (**Supplementary Table S2**). As a prerequisite to identify associations, respective markers need to be in a polymorphic state within the TCCC. The ratio of polymorphic markers within the Top10_MTAs differed between the sampling strategies (**Supplementary Tables S5**, **S6**). As expected, more Top10_MTAs markers appeared polymorphic with an increasing size of the TCCC and restrictions in the ratio of polymorphic markers were only present in TCCCs of smaller sizes. While sampling strategy 1T_rank resulted in the overall lowest ratio of polymorphic markers and the sampling strategies 2T_rank, 2T_Gdiv&Gsim and 2T_Gdiv&Gdiv prevented such restriction for all sizes of TCCCs and both traits.

**Figure 6:**
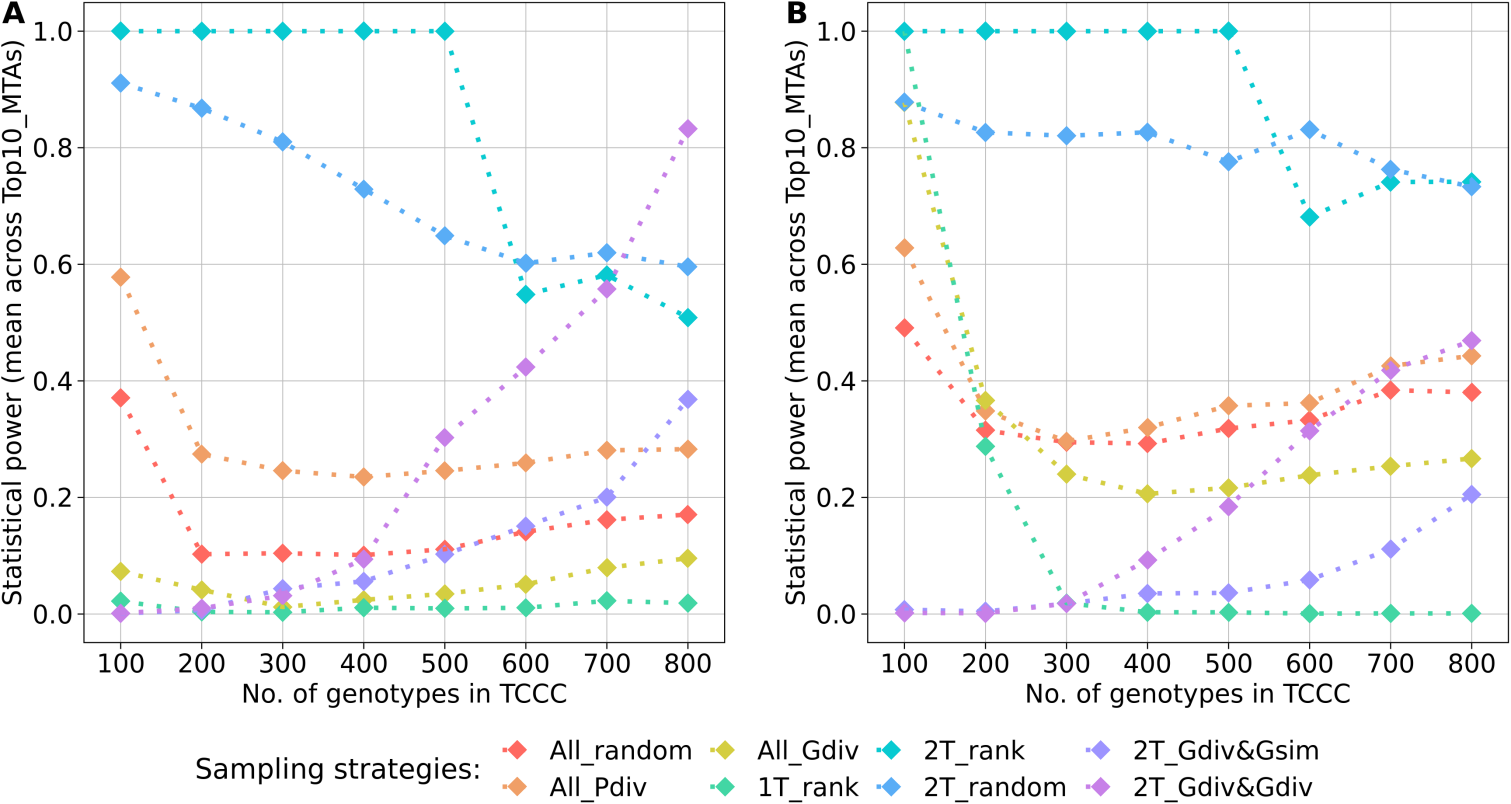
Arithmetic mean of the statistical power for the identification of marker-trait-associations calculated based on 10 markers of the Top10_MTAs panel. Power estimates were performed within trait-customized core collections for yellow rust susceptibility **(A)** and stem lodging **(B)**, respectively. Values are depicted for eight different sampling strategies representing mean values of 50 independent replications. For the calculation of the mean, the estimated power of monomorphic markers was considered with a value of zero. The estimated power of a marker was excluded if the proportion of the explained phenotypic variance could not be estimated within a specific trait-customized core collection.

A key parameter of the comparison was the statistical power for the identification of the Top10_MTAs markers. For both traits, the average power across these markers followed a general pattern (**Figure 6**). Highest power resulted from sampling opposite phenotypic extremes without considering the genotypic information - strategies 2T_rank and 2T_random. Sampling strategies which rely on phenotypic as well as genotypic information showed strong increase in the power with increasing numbers of accession samples. Random sampling (All_random) and sampling from the entire phenotypic range (All_Pdiv) led to moderate high statistical power for 100 accession samples, followed by a strong drop when increasing to 200 accession samples but which did not alter much with increasing sizes of TCCCs further. Sampling only accessions with beneficial phenotypes (1T_rank) predominantly resulted in TCCCs with unfavorable power estimates: the power decreased from a peak value at a TCCC size of 100 with increasing sizes of TCCCs for SL, the power remained in this case constantly low for YR. For TCCCs of smaller size, low values of power might also originate from the fact that the monomorphic state of a marker results in a power of zero and thus, conclusions based on single markers can be diverging from the general trend. Additionally, the proportion of the explained phenotypic variance could not be calculated for some markers within the TCCC for factors such as complete collinearity and thus, power estimates were excluded in this specific case.

Investigation of statistical power separately per marker was dominated by strong fluctuation. As a general trend, the findings described for the averaged power (**Figure 6**) were also found for the single marker of the Top10_MTAs for SL (**Supplementary Figure S3**) and for YR (**Supplementary Figure S4**). Contradicting to the general assumption, decreasing power estimates were found for increasing TCCC sizes for the strategies 2T_rank and T_random in combination with several markers. For SL, the marker-specific MAF of six markers among the Top10_MTAs was relatively high regardless of the sampling which indicate that they are not associated with rare alleles *per se* (**Supplementary Figure S5**). For YR, low MAF could be observed for multiple markers among the Top10_MTAs regardless of the sampling strategy as well as the size of the TCCC (**Supplementary Figure S6**). One marker even showed a maximum of MAF below 5% for all combination of sampling strategy and sizes of the TCCC (4A_725504657). These rare variants risk to fall below a MAF-threshold which is typically applied in GWAS studies and thus, might not be detectable.

## 4 Discussion

The presented comparison of eight strategies to sample a TCCC aimed to provide guidance for improved scrutinization of genebank collections by engaging GWAS. The results suggest that not a single approach can be optimally suited for all traits; however, some strategies, such as 1T_rank, showed generalized disadvantages. In consequence, the choice for a selection strategy is guided by a trade-off between the presented six evaluated criteria. Undoubtedly, the estimated statistical power, as defined by Wang and Xu (2019), is a corner stone of evaluating selection strategies which should later be analyzed engaging GWAS. Nevertheless, a mere focus on statistical power might allow for genetic redundancies in the TCCC and fortify the impact of population stratification. Thus, a case-dependent weighing of criteria is needed.

### 4.1 Phenotypic contrast boosts power in association studies

Sampling extreme phenotypes for association studies has been reported to leverage detection power (Van Gestel et al., 2000; Xing and Xing, 2009) and similarly, the presented results show an increase in estimated power for TCCCs by incorporating phenotypic extremes (**Figure 6**). As demonstrated by the comparison of 1T_rank with 2T_rank for YR, the contrast between positive and negative tails is in particular important for GWAS; thus, a mere accumulation of possible desired donor genotypes is hardly effective in this respect. In addition, the variance components used for the calculation had a strong impact: particularly for SL, 2T_rank and 2T_random combined high genetic and low residual variances (**Supplementary Figures S7**, **S8**), while 2T_Gdiv&Gsim and 2T_Gdiv&Gdiv resulted in the opposite. Due to the minimization of Rogers’ distances between the positive and negative tails in the latter two sampling strategies, variation is assigned as residual variance even though the genetic diversity was maximized within the positive tail. This finding highlights the importance of the relative magnitude of polygenic variance compared with the residual variance. The ratio of polygenic variance to the residual variance is given by λ in Equation 2. The larger the value for λ gets, the greater the non-centrality parameter δ can become and as a consequence, a high statistical power could be expected. Representing phenotypic extremes could result in an enlarged genetic variance and thus in an elevated polygenic contribution to the phenotypic variance. Therefore, one can conclude that data with high genomic heritability has the potential for high statistical power in GWAS.

### 4.2 Limitations for the identification of rare variants within TCCCs

The identification of rare variants is a well-known problem in GWAS (Myles et al., 2009; Kosmicki et al., 2016) and grouping of genotypes was proposed as a method to increase the frequency of rare variants (Kosmicki et al., 2016). In the present study, sampling extreme phenotypes was implemented following the assumption that accessions with advantageous phenotypes have genotypes that are enriched with rare beneficial variants. In particular for SL, trends confirmed this assumption because the average MAF of the markers in the Top10_MTAs panel were augmented by the strategy 1T_rank, but also mildly augmented by the strategies 2T_rank and 2T_random, compared to All_random (**Supplementary Figure S9**). This trend was however not similarly present for YR and may even revert to the opposite at the level of individual Top10_MTAs markers, like for instance in case of marker 7A_367972613 (**Supplementary Figure S5**). A possible explanation could be the contrasting underlying genetic architectures assumed from the Gaussian-like (YR) and L-shaped (SL) distributions. Based on two disease traits in humans, Xing and Xing (2009) investigated the effect of extreme phenotype sampling on GWAS. The authors reported that common variants benefit in particular from this strategy and less prominent effect was reported on rare variants. This could arguably be the case for some of the markers in the Top10_MTAs panel in the present study.

Assuming that a targeted enrichment for rare variants would be difficult for specific traits, a selection strategy increasing the overall MAF could have its merits to avoid problems joint with rare associated variants. For both traits, sampling a TCCC with All_Gdiv clearly increased the overall mean MAF compared to the other sampling strategies (**Supplementary Figure S10**). The selected accession samples would not be specifically optimized for one trait but rather suits for all traits moderately. On the other hand, such a general approach would demand extremely large TCCC sizes, arguably larger than the sizes tested in the present report, in order to allow for a suitable statistical power and therefore, remains rather theoretical given the limitation in funding and capacities often-faced by genebank institutions.

In addition to the MAF, the effect sizes of a variant determine the success of the identification in GWAS (Kosmicki et al., 2016; Wang and Xu, 2019). Uncommon variants of large or at least medium effect size in the total collection are the only reasonable target of a GWAS in order to justify the high costs of creating and deeply characterizing a TCCC. When focusing on such markers across the Top10_MTAs panel (e.g. YR: 2B_57683037, 6A_135235117; SL: 2D_186808096), the power estimates per sampling strategy revealed that sampling from the phenotypic extremes based on the rank (2T_rank) outcompetes the other sampling strategies (**Supplementary Figures S3**, **S4**). The differences between 2T_rank and 2T_random were however small in these cases. It should be mentioned that a drop in estimated power was observed for the sampling strategy 2T_rank in combination with larger TCCCs for some markers with low effect sizes (e.g. YR: 4A_725504657; SL: 7A_367972613) (**Supplementary Figures S3**, **S4**). While the sampling strategy 2T_rank showed higher power only for some particular TCCC sizes for variants with a low effect size, strategies which did not rely on the phenotypic extremes (All_random, All_Pdiv and All_Gdiv) showed an overall low performance in these cases. Similarly, Wang and Xu (2019) found an asymptotic increase of estimated power when increasing the effect size of a simulated QTL in a hybrid population as well as in a set of recombinant inbred lines of rice. These findings highlight that rather the effect size of a variant determines the success of GWAS than the composition of the TCCC. Identification of small effect variants cannot be a predictable aim of creating a TCCC; they will probably remain unnoticed.

### 4.3 Reduction of population stratification by maximizing genetic diversity

Sampling a TCCC exclusively from the phenotypic extremes increases the magnitude of population stratification exceeding the base level present in the genebank collection. For GWAS in general, this concern was already raised by Guey and collaborators (Guey et al., 2011) and later proven based on simulations by Panarella and Burkett (2019) who reported on a substantial increase in false-discovery rate compared with random sampling. The presented real data scenario is in accordance with these previous findings. Sampling from the two phenotypic extremes, specifically based on the rank (2T_rank), forces a boost in the correlation between phenotype and genotype when compared to the random sampling which is a stable representation of the characteristics of the entire population (**Figure 3**). Two options of accounting for population stratification were presented here; either minimize the phenotypic diversity (1T_rank) or maximize the genetic diversity (2T_Gdiv&Gsim, 2T_Gdiv&Gdiv and All_Gdiv). While the first option leads to a low averaged power estimate for the Top10_MTAs panel (**Figure 6**), the latter option seems to be vital to subdue population stratification in genebank genomics.

### 4.4 Maximizing genetic diversity avoids less informative duplicates

Maximizing the genetic diversity within the TCCC is negatively associated with the number of duplicate genotypes accumulated (**Figure 4**). As mentioned earlier, duplicate genotypes occur widespread across genebank collections proven for many institutes and crops (FAO, 2010). Without any doubts, the identification of completely identical duplicate genotypes is difficult due to the simplifying nature of marker data and therefore, the definition of a threshold value for similarity will always be disputable. Moreover, the threshold value in the present study might not only include identical but also highly similar genotypes. In theory, including completely identical genotypes within a collection is costly with respect to maintaining an additional accession in a pure and safe manner without gaining additional information for breeding and research. Therefore, discussions on removing duplicates from the collection could be a long-term perspective for easier exploitation of genebanks, expressly including the development of TCCCs. However, including duplicates in TCCCs is in particular an even more substantial waste of resources. In the first years after establishment of the TCCC, costs will be high due to more thorough genotyping, such as whole-genome-sequencing, phenotyping with more replications or at more locations, and the creation of crosses with elite germplasm. Duplicates will however be in every respect less informative for pre-breeding purposes. Additionally, TCCCs are only a meaningful contribution to the exploration of genebank collections if preserved on at least a medium-term and made publicly available to breeders without considerable burdens. For both duplicates and unique accessions, these costs incur continuously year after year and are probably often underestimated in the initial funding. Moreover, duplicate genotypes could increase the MAF of rare associated variants in GWAS without improving the resolution for the specific QTL due to strong linkage within haplotypes. Finally, having approximately or exactly duplicated columns and rows in the matrix used for kinship correction in GWAS could increase collinearity issues during mixed model computation. Already at the early times of creating CCs, Brown (1989b) stated that the sampling of core collections should not tolerate redundant entries. Nowadays, the identification of such accessions is more accurate having genomic data at hand and genebank genomics should take advantage of this.

### 4.5 Impact and considerations of a suitable size of a TCCC

The size of a TCCC is the key variable affecting all parameters discussed above and no size was determined leading to an adequate optimization of all the parameters simultaneously. However, the benefits of sampling strategies were associated with certain ranges of TCCC sizes. As a general trend, sampling strategies which do not rely on any genotypic information do not benefit of larger sizes of TCCC. Especially, large-effect variants can be identified within small TCCCs of just 100 accession samples if the phenotypic contrast is maximized. With a TCCC size of about ≥8% of the entire collection (corresponding to ≥500 accession samples), duplicated genotypes are getting accumulated in the TCCC (**Figure 4**) while the statistical power is stagnating or even decreasing (**Figure 6**). In contrast, sampling strategies engaging an algorithm for genetic maximization allow for the incorporation of more accession samples into the TCCC. Up to 11% of all accession samples can be selected without accumulating many duplicated genotypes within the TCCC for both traits (**Figure 4**). These additional accessions could allow to charge the TCCC with more beneficial variants at higher frequency. While increasing the TCCC size to the aforementioned maximum is favorable with respect to the constantly increasing statistical power found for 2T_Gdiv&Gsim and 2T_Gdiv&Gdiv, smaller TCCC sizes should preferably be chosen to account for population stratification (**Figure 3**).

Utilizing simulated data in research allows for a targeted alteration of a single parameter. As a consequence, dependency of statistical power on the number of genotypes was found to be very clear and only increasing trends were detected by Wang and Xu (2019). In contrast, the present real-data scenarios demonstrated that additional genotypes can severely change the characteristics of a GWAS population, such as the variance component for the genotype (**Supplementary Figure S7**), and may even lead to reverting trends for the statistical power (**Figure 6**). Considering the complexity faced in genebank genomics with broad genetic diversity, these unexpected deviations from assumed trends should be considered in future.

The presented study demonstrates opportunities and obstacles in selecting a TCCC specifically for the identification of donor genotypes based on the example of a relatively large wheat germplasm collections at the IPK Genebank. In practice, much smaller or genetically narrower genebank collections may require different approaches. In general, the selection strategy will largely depend on the availability of phenotypic and genotypic data as well as the present resources to gather more data in advance for the sampling of a TCCC. As a clear guidance for genebank curators we can summarize that knowing the phenotypic distribution is most important and will indicate the possible scope of a later GWAS. TCCC should be interpreted as a top-down strategy: a large-scale phenotypic evaluation using cheaper, however mostly less accurate, methods would be a suitable start before creating a TCCC. For most genebanks, this might rely on the curation of historical records originating from past field trials or taken during seed multiplication. If a TCCC of moderate size is intended, sampling accessions with extremely contrasting phenotypes might be most promising. In most genebanks, genotypic data might not be at hand for entire collections and relying on genotypic information for the sampling strategy will increase the costs tremendously. However, TCCCs of large size will especially profit of strategies such as 2T_Gdiv&Gdiv in order to avoid genetic redundancies. Once a TCCC has been established for one trait, a shrewd strategy for the enlargement considering additional traits is the following step. Depending on the selection strategy, the ratio of accessions samples overlapping between TCCCs could help to reduce costs for the leveraging of genebank collections for many traits in future.

## Data Availability

The marker profiles of the analyzed accession samples were previously published (Schulthess et al., 2022a, b) and can be found online. Phenotypic records for YR originate from recent work by Schulthess and collaborators (Schulthess et al., 2022a, b).
