# Supplementary material for: Trait-customized sampling of core collections from a winter wheat genebank collection supports association studies
Source: Front Plant Sci. 2024 Oct 2;15:1451749. doi: 10.3389/fpls.2024.1451749 (PMC11479895; doi:10.3389/fpls.2024.1451749)
Supplement: Supplementary file 1 [file DataSheet1.pdf]

Supplementary Material

1 Supplementary Figures and Tables

1.1 Supplementary Tables

Supplementary Table 1: Summary statistics for the two traits yellow rust susceptibility and stem lodging. Given are the estimated variance components for tested entries ($\hat{\boldsymbol{\sigma}}_{\boldsymbol{g}}^{\mathbf{2}}$), their interaction with experiments ($\hat{\boldsymbol{\sigma}}_{\boldsymbol{ge}}^{\mathbf{2}}$) and residual effect ($\hat{\boldsymbol{\sigma}}_{\boldsymbol{res}}^{\mathbf{2}}$), the average number of effective replicates per entry (**No. rep.)**, the average number of phenotyping experiments (**No. exp.**), the heritability estimates (***h***^{2}), the number of tested entries and the number of accessions samples with complete information including best linear unbiased estimates (**BLUE**) and marker profiles from genotyping-by-sequencing (**GBS**).

**Trait**	$\hat{\boldsymbol{\sigma}}_{\boldsymbol{g}}^{\mathbf{2}}$	$\hat{\boldsymbol{\sigma}}_{\boldsymbol{ge}}^{\mathbf{2}}$	$\hat{\boldsymbol{\sigma}}_{\boldsymbol{res}}^{\mathbf{2}}$	**No. rep.**	**No. exp.**	$\boldsymbol{h}^{2}$	**No. entries**	**Acc. samples with BLUE & GBS**
Yellow rust susceptibility	1.34	0.31	0.77	1.91	2.36	0.82	7,762	6,300
Stem lodging	2.98	0.71	1.03	1.91	2.55	0.86	7,745	6,251

Supplementary Table 2: Information about the 10 markers of each Top10_MTAs panel for the traits yellow rust susceptibility and stem lodging. Depicted are the marker names, the -$\log_{10}(p)$-values originating from genome-wide association studies, the effect of each marker on the trait (***r^2***), the minor-allele-frequency (**MAF**) as well as a summary based on marker effect (high: ***r^2*** > 0.01, medium: ***r^2*** > 0.001, low: ***r^2*** > 0.0001) and the frequency (common: **MAF** > 0.05, rare: **MAF** > 0.01). *: Due to complete collinearity with marker 2D_186808096, the marker conveyed redundant information and the ***r^2*** could not be computed.

Trait	Marker name	-$\log_{10}(p)$-value	r^2	MAF	effect	frequency
Yellow rust susceptibility	2A_652605864	5.9535	0.0135	0.0994	high	common
	6A_135235117	4.8075	0.0109	0.0387	high	Rare
	2B_57683037	5.5971	0.0205	0.011	high	Rare
	5B_617809290	5.0993	0.005	0.1909	medium	common
	5B_536040546	8.0334	0.0013	0.1643	medium	common
	5B_501480761	14.2644	0.0012	0.0933	medium	common
	5A_461316244	4.7704	0.0018	0.0308	medium	Rare
	4B_172322427	8.224	0.0002	0.1145	low	common
	5B_515186726	13.3986	0.0002	0.0887	low	common
	4A_725504657	5.2302	0.0007	0.0183	low	Rare
Stem lodging	3B_629550543	9.4709	0.0157	0.2193	high	common
	6A_601254786	5.84	0.0334	0.0432	high	common
	2D_186808122*	5.1034	-	0.0209	high	Rare
	2D_186808096	5.1034	0.0447	0.0209	high	Rare
	5B_590614570	5.489	0.0072	0.3347	medium	common
	5A_474478881	9.7265	0.0038	0.2884	medium	common
	1B_512816125	7.1172	0.0073	0.224	medium	common
	3B_756626133	6.0518	0.0004	0.202	low	common
	2A_700367669	7.267	0.0009	0.1885	low	common
	7A_367972613	6.1492	0.0008	0.0734	low	common

Supplementary Table 3: Effective population size [N_e] of trait-customized core collections for the trait yellow rust susceptibility derived from eight distinct sampling strategies. Per sampling strategy, values are presented for eight different sizes of trait-customized core collections, ranging from 100 to 800 accession samples. Each value is the arithmetic mean of 50 independent replications; the standard deviations are shown in brackets.

	Sampling strategy							
**Size**	**All_random**	**All_Pdiv**	**All_Gdiv**	**1T_rank**	**2T_rank**	**2T_random**	**2T_Gdiv& Gsim**	**2T_Gdiv& Gdiv**
100	54.20 (σ = 9)	58.84 (σ = 6.78)	309.77 (σ = 21.59)	42.21 (σ = 0)	39.47 (σ = 0)	41.25 (σ = 4.76)	73.53 (σ = 1.47)	86.92 (σ = 1.23)
200	68.97 (σ = 7.99)	75.03 (σ = 8.46)	354.03 (σ = 21.73)	42.70 (σ = 0)	55.51 (σ = 0)	53.89 (σ = 4.59)	85.73 (σ = 1.21)	105.17 (σ = 2.42)
300	77.18 (σ = 7.55)	84.07 (σ = 5.6)	360.73 (σ = 14.73)	52.50 (σ = 0)	62.23 (σ = 0)	63.39 (σ = 4.31)	90.25 (σ = 0.89)	111.20 (σ = 1.01)
400	85.80 (σ = 6.78)	90.87 (σ = 6.93)	338.72 (σ = 24.24)	61.33 (σ = 0)	74.17 (σ = 0)	70.98 (σ = 3.84)	96.45 (σ = 0.83)	118.05 (σ = 0.85)
500	89.95 (σ = 6.04)	95.78 (σ = 5.13)	319.61 (σ = 24.03)	65.93 (σ = 0)	77.45 (σ = 0)	76.66 (σ = 3.55)	101.14 (σ = 0.45)	124.75 (σ = 1.15)
600	96.96 (σ = 6.15)	98.33 (σ = 4.54)	305.93 (σ = 12.35)	70.72 (σ = 0)	77.37 (σ = 0)	81.04 (σ = 2.64)	100.69 (σ = 0.63)	126.44 (σ = 1.25)
700	99.01 (σ = 5.19)	101.87 (σ = 5.38)	298.89 (σ = 5.75)	75.21 (σ = 0)	81.09 (σ = 0)	84.62 (σ = 3.32)	101.56 (σ = 0.41)	127.21 (σ = 1.03)
800	102.84 (σ = 4.79)	103.91 (σ = 4.08)	294.19 (σ = 5.59)	82.46 (σ = 0)	82.05 (σ = 0)	88.18 (σ = 3.07)	100.61 (σ = 0.21)	116.74 (σ = 0.55)

Supplementary Table 4: Effective population size [N_e] of trait-customized core collections for the trait stem lodging derived from eight distinct sampling strategies. Per sampling strategy, values are presented for eight different sizes of trait-customized core collections, ranging from 100 to 800 accession samples. Each value is the arithmetic mean of 50 independent replications; the standard deviations are shown in brackets.

	Sampling strategy							
**Size**	**All_random**	**All_Pdiv**	**All_Gdiv**	**1T_rank**	**2T_rank**	**2T_random**	**2T_Gdiv& Gsim**	**2T_Gdiv& Gdiv**
100	53.12 (σ = 7.77)	58.54 (σ = 8.51)	305.64 (σ = 24.44)	70.62 (σ = 0)	68.99 (σ = 0)	54.54 (σ = 6.48)	128.48 (σ = 4.49)	115.86 (σ = 5.07)
200	68.42 (σ = 7.15)	68.57 (σ = 6.6)	348.93 (σ = 22.88)	76.63 (σ = 0)	79.63 (σ = 0)	68.80 (σ = 6.57)	155.32 (σ = 4.05)	162.64 (σ = 2.56)
300	78.30 (σ = 6.89)	76.76 (σ = 5.81)	355.41 (σ = 18.61)	83.37 (σ = 0)	80.63 (σ = 0)	79.06 (σ = 6.17)	158.73 (σ = 1.95)	177.82 (σ = 2.79)
400	85.23 (σ = 6.71)	80.29 (σ = 5.15)	338.96 (σ = 28.41)	92.06 (σ = 0)	82.77 (σ = 0)	84.83 (σ = 5.5)	161.96 (σ = 1.3)	192.14 (σ = 1.49)
500	91.85 (σ = 6.18)	84.71 (σ = 4.2)	311.94 (σ = 17.55)	99.24 (σ = 0)	87.10 (σ = 0)	89.59 (σ = 4.87)	158.89 (σ = 1.44)	188.64 (σ = 1.11)
600	97.30 (σ = 6.91)	87.88 (σ = 4.72)	304.56 (σ = 11.05)	105.34 (σ = 0)	92.90 (σ = 0)	92.27 (σ = 4.21)	150.22 (σ = 0.65)	173.16 (σ = 1.06)
700	99.77 (σ = 6.53)	89.83 (σ = 3.52)	300.04 (σ = 8.07)	112.41 (σ = 0)	92.12 (σ = 0)	98.07 (σ = 4.7)	141.18 (σ = 0.72)	154.9 (σ = 2.33)
800	103.17 (σ = 5.06)	92.03 (σ = 3.72)	293.86 (σ = 5.32)	117.59 (σ = 0)	95.87 (σ = 0)	99.09 (σ = 3.13)	133.48 (σ = 0.31)	135.05 (σ = 0.58)

Supplementary Table 5: Average number of in total 10 markers of the Top10_MTAs panel which are in a polymorphic state within the trait-customized core collections for the trait yellow rust susceptibility. The analyzed samples of genotypes resulted from eight distinct sampling strategies which were applied for eight different sizes of trait-customized core collections, ranging from 100 to 800 accession samples. Each value is the arithmetic mean of 50 independent replications; the standard deviations are shown in brackets.

	**Sampling strategy**							
**Size**	**All_random**	**All_Pdiv**	**All_Gdiv**	**1T_rank**	**2T_rank**	**2T_random**	**2T_Gdiv& Gsim**	**2T_Gdiv& Gdiv**
100	9.46 (σ = 0.61)	9.88 (σ = 0.39)	9.74 (σ = 0.44)	8.00 (σ = 0)	10.00 (σ = 0)	9.50 (σ = 0.65)	10.00 (σ = 0)	10.00 (σ = 0)
200	9.78 (σ = 0.55)	10.00 (σ = 0)	10.00 (σ = 0)	8.00 (σ = 0)	10.00 (σ = 0)	9.88 (σ = 0.33)	10.00 (σ = 0)	10.00 (σ = 0)
300	9.94 (σ = 0.24)	10.00 (σ = 0)	10.00 (σ = 0)	9.00 (σ = 0)	10.00 (σ = 0)	9.98 (σ = 0.14)	10.00 (σ = 0)	10.00 (σ = 0)
400	9.98 (σ = 0.14)	10.00 (σ = 0)	10.00 (σ = 0)	9.00 (σ = 0)	10.00 (σ = 0)	10.00 (σ = 0)	10.00 (σ = 0)	10.00 (σ = 0)
500	10.00 (σ = 0)	10.00 (σ = 0)	10.00 (σ = 0)	10.00 (σ = 0)	10.00 (σ = 0)	10.00 (σ = 0)	10.00 (σ = 0)	10.00 (σ = 0)
600	10.00 (σ = 0)	10.00 (σ = 0)	10.00 (σ = 0)	10.00 (σ = 0)	10.00 (σ = 0)	10.00 (σ = 0)	10.00 (σ = 0)	10.00 (σ = 0)
700	10.00 (σ = 0)	10.00 (σ = 0)	10.00 (σ = 0)	10.00 (σ = 0)	10.00 (σ = 0)	10.00 (σ = 0)	10.00 (σ = 0)	10.00 (σ = 0)
800	10.00 (σ = 0)	10.00 (σ = 0)	10.00 (σ = 0)	10.00 (σ = 0)	10.00 (σ = 0)	10.00 (σ = 0)	10.00 (σ = 0)	10.00 (σ = 0)

Supplementary Table 6: Average number of in total 10 markers of the Top10_MTAs panel which are in a polymorphic state within the trait-customized core collections for the trait stem lodging. The analyzed samples of genotypes resulted from eight distinct sampling strategies which were applied for eight different sizes of trait-customized core collections, ranging from 100 to 800 accession samples. Each value is the arithmetic mean of 50 independent replications; the standard deviations are shown in brackets.

	**Sampling strategy**							
**Size**	**All_random**	**All_Pdiv**	**All_Gdiv**	**1T_rank**	**2T_rank**	**2T_random**	**2T_Gdiv& Gsim**	**2T_Gdiv& Gdiv**
100	9.64 (σ = 0.78)	10.00 (σ = 0)	10.00 (σ = 0)	8.00 (σ = 0)	10.00 (σ = 0)	9.84 (σ = 0.51)	10.00 (σ = 0)	10.00 (σ = 0)
200	10.00 (σ = 0)	10.00 (σ = 0)	10.00 (σ = 0)	8.00 (σ = 0)	10.00 (σ = 0)	10.00 (σ = 0)	10.00 (σ = 0)	10.00 (σ = 0)
300	10.00 (σ = 0)	10.00 (σ = 0)	10.00 (σ = 0)	8.00 (σ = 0)	10.00 (σ = 0)	10.00 (σ = 0)	10.00 (σ = 0)	10.00 (σ = 0)
400	10.00 (σ = 0)	10.00 (σ = 0)	10.00 (σ = 0)	10.00 (σ = 0)	10.00 (σ = 0)	10.00 (σ = 0)	10.00 (σ = 0)	10.00 (σ = 0)
500	10.00 (σ = 0)	10.00 (σ = 0)	10.00 (σ = 0)	10.00 (σ = 0)	10.00 (σ = 0)	10.00 (σ = 0)	10.00 (σ = 0)	10.00 (σ = 0)
600	10.00 (σ = 0)	10.00 (σ = 0)	10.00 (σ = 0)	10.00 (σ = 0)	10.00 (σ = 0)	10.00 (σ = 0)	10.00 (σ = 0)	10.00 (σ = 0)
700	10.00 (σ = 0)	10.00 (σ = 0)	10.00 (σ = 0)	10.00 (σ = 0)	10.00 (σ = 0)	10.00 (σ = 0)	10.00 (σ = 0)	10.00 (σ = 0)
800	10.00 (σ = 0)	10.00 (σ = 0)	10.00 (σ = 0)	10.00 (σ = 0)	10.00 (σ = 0)	10.00 (σ = 0)	10.00 (σ = 0)	10.00 (σ = 0)

1.2 Supplementary Figures

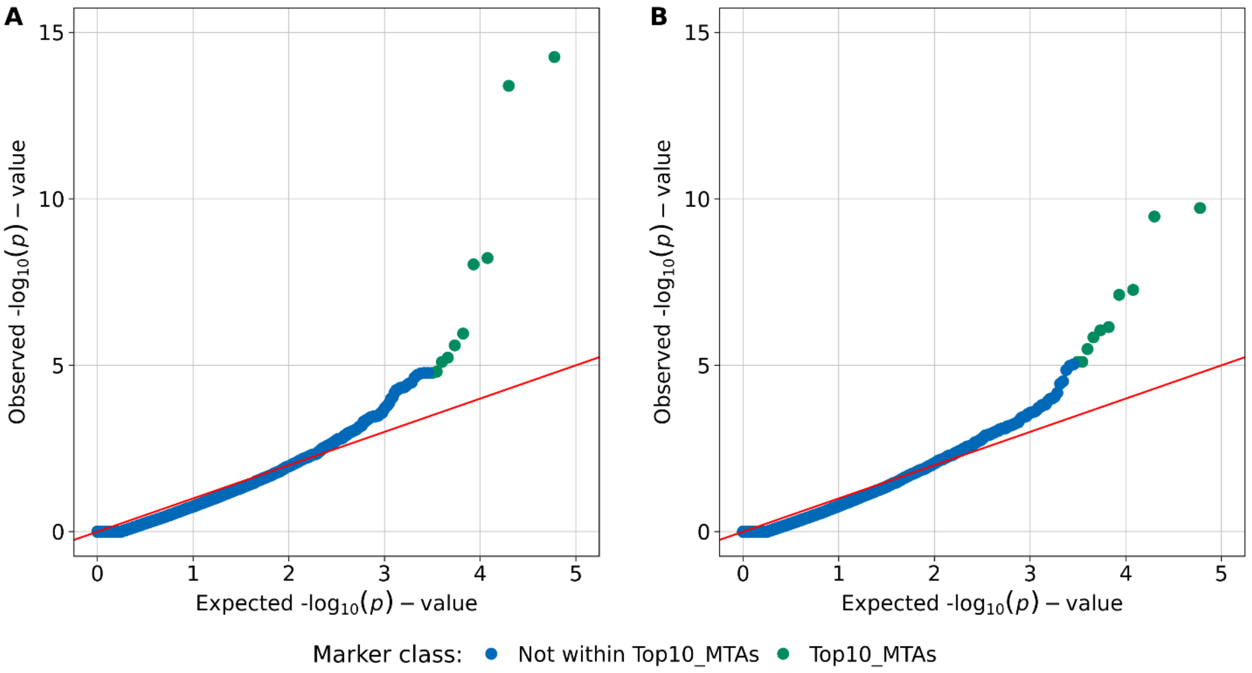

Supplementary Figure 1: Quantile-quantile-plots based on the -log$_{10}$(p)-values originating from genome-wide association studies for the traits yellow rust susceptibility (A) and stem lodging (B). Plotted are the observed -log$_{10}$(p)-values for all markers against the values expected based on a normal distribution. The red curve displays the assumption that the observed -log$_{10}$(p)-values would perfectly follow the expectation. The color of the markers indicates if they were incorporated in the Top10_MTAs marker panel.

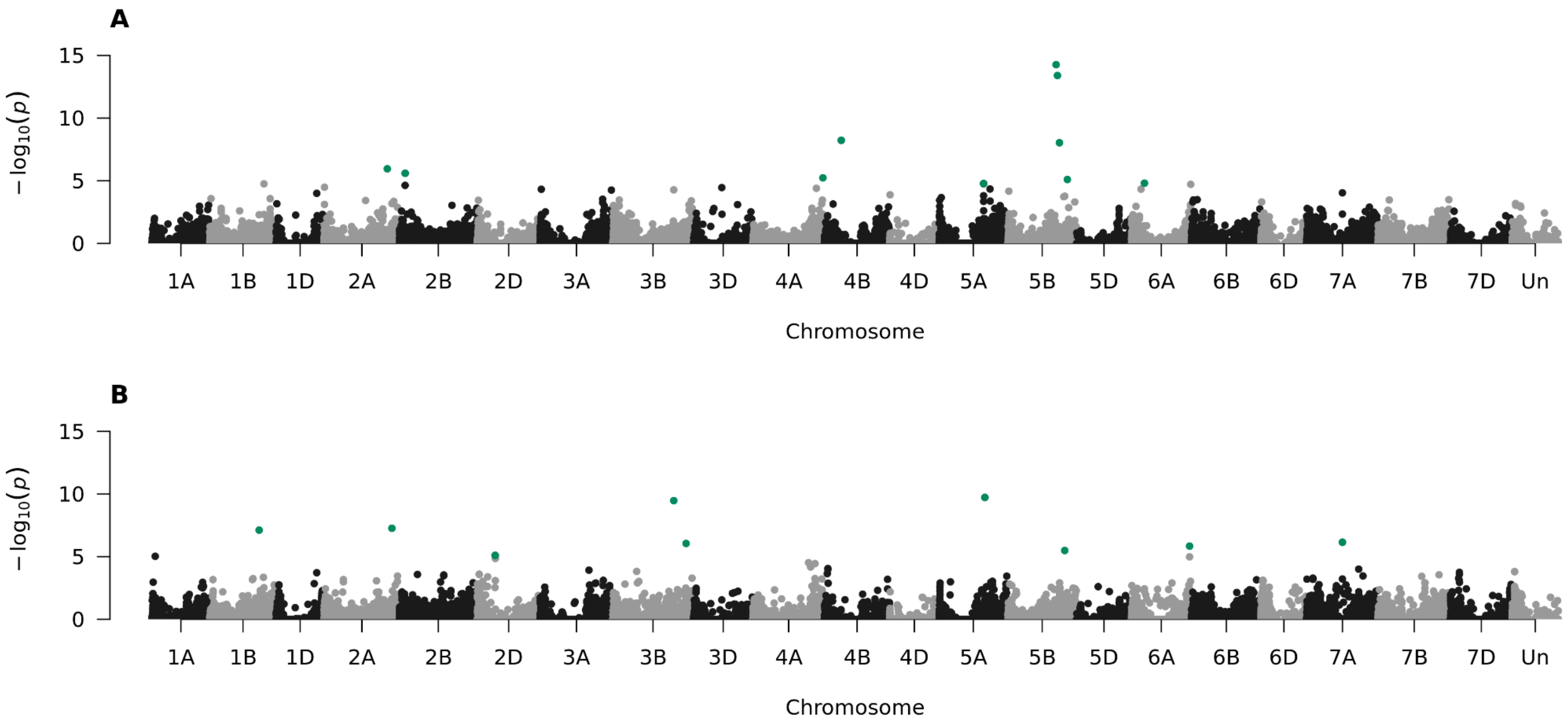

Supplementary Figure 2: Manhattan plots depicting the results of the genome-wide association studies for the traits yellow rust susceptibility (A) and stem lodging (B). Plotted are the -$\log_{10}(p)$-values for the association with the trait for markers located on the 21 chromosomes and for a group of markers which could not be assigned with certainty to a single chromosome (Un). Markers of the Top10_MTAs panels are highlighted in green.

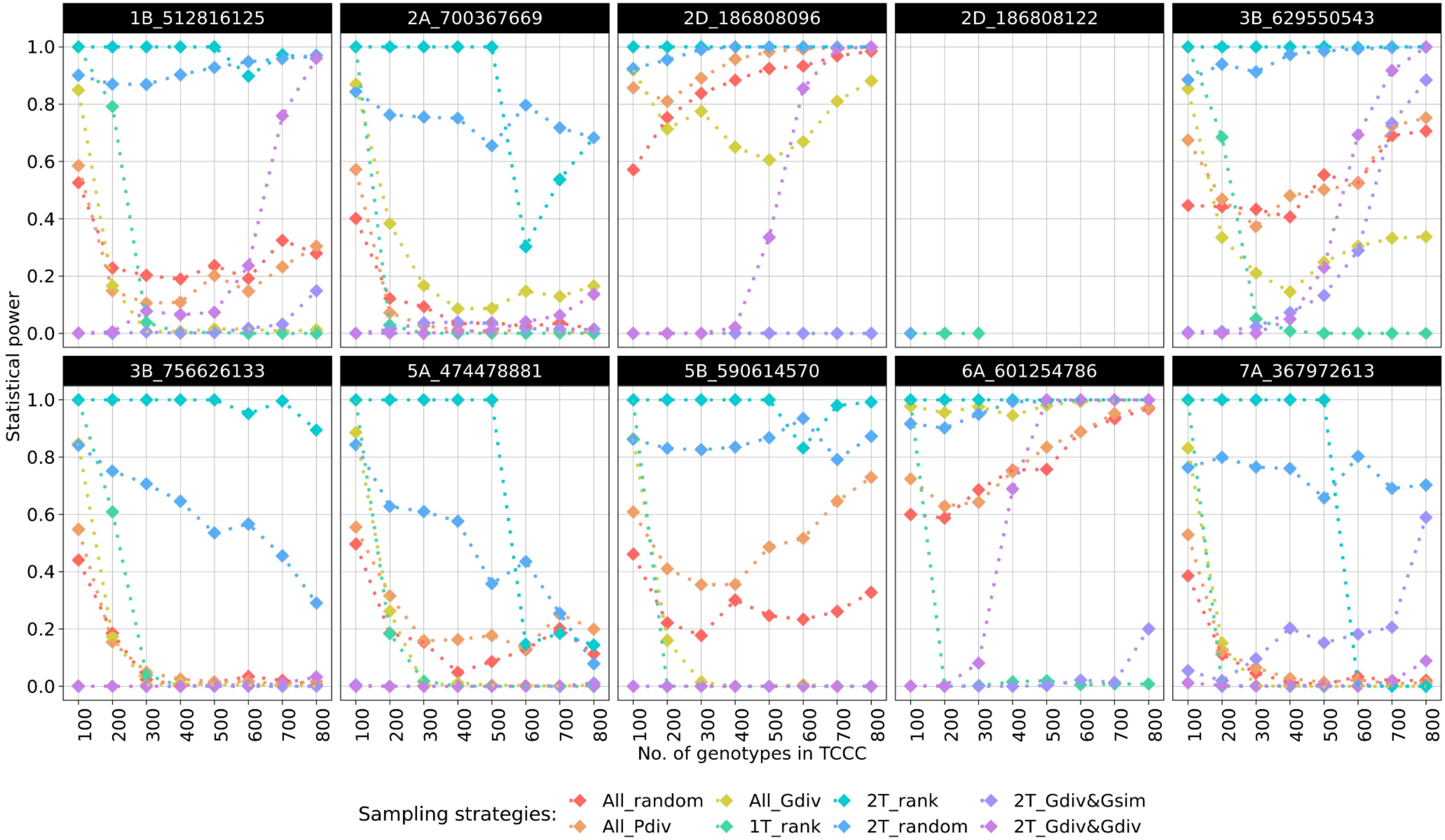

Supplementary Figure 3: Statistical power for the identification of marker-trait-associations for each of the 10 markers of the Top10_MTAs panel for the trait stem lodging depending on the size of the trait-customized core collection. Values are depicted for eight different sampling strategies representing mean values of 50 independent replications. For the calculation of the mean, the estimated power of monomorphic markers was considered with a value of zero. The estimated power of a marker was excluded if the proportion of the explained phenotypic variance could not be estimated within a specific trait-customized core collection.

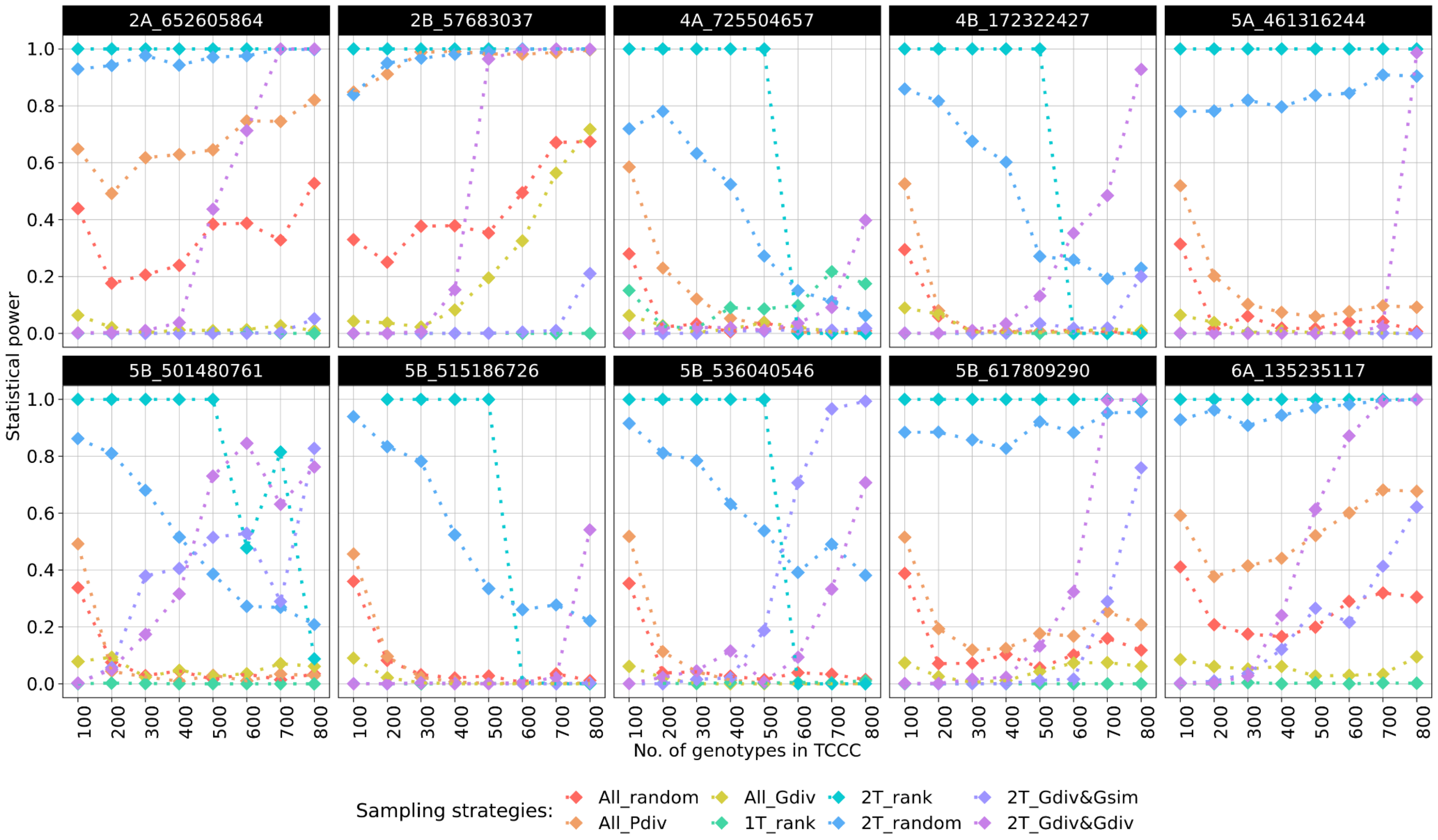

Supplementary Figure 4: Statistical power for the identification of marker-trait-associations for each of the 10 markers of the Top10_MTAs panel for the trait yellow rust susceptibility depending on the size of the trait-customized core collection. Values are depicted for eight different sampling strategies representing mean values of 50 independent replications. For the calculation of the mean, the estimated power of monomorphic markers was considered with a value of zero. The estimated power of a marker was excluded if the proportion of the explained phenotypic variance could not be estimated within a specific trait-customized core collection.

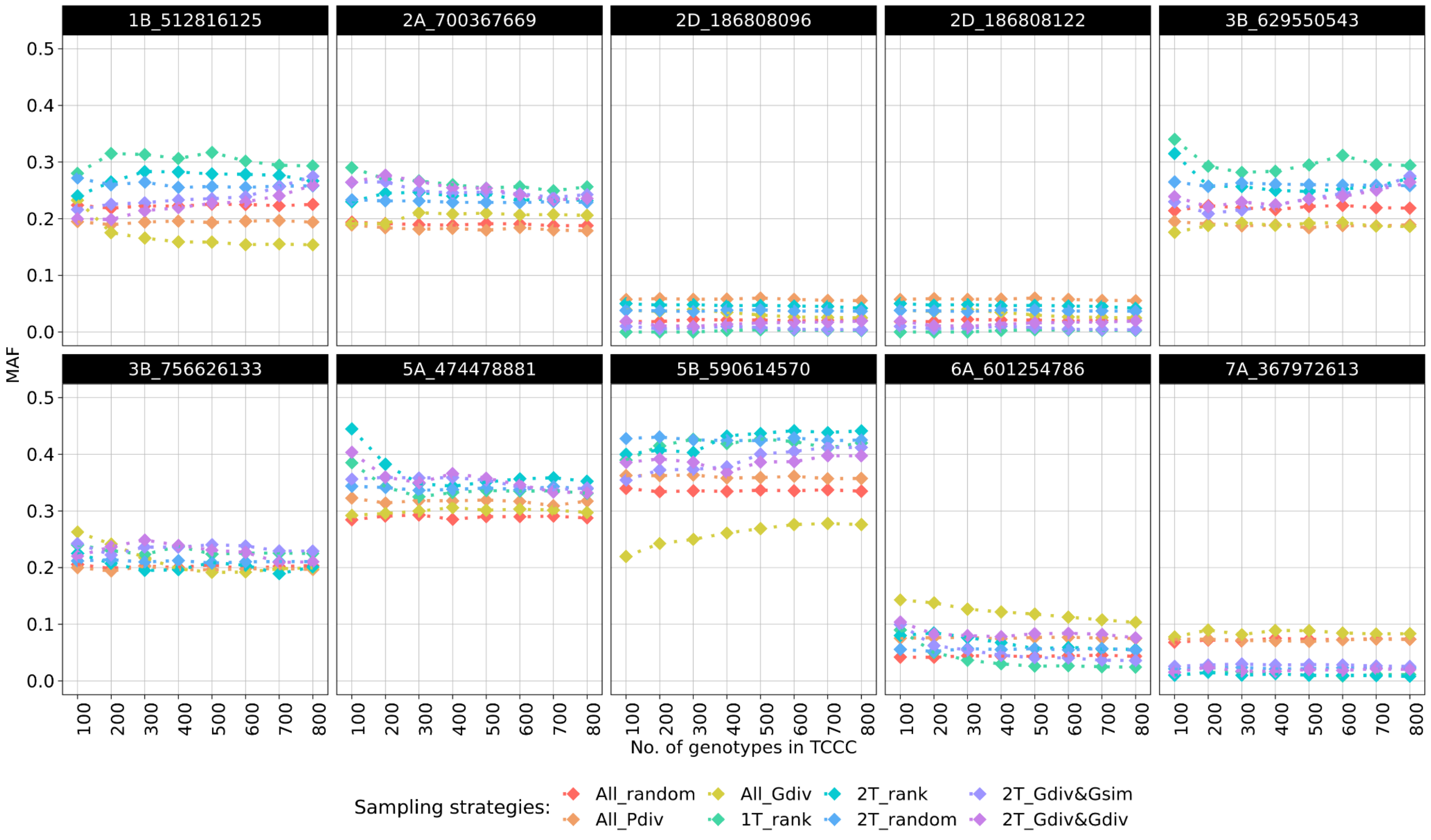

Supplementary Figure 5: Minor-allele-frequency (MAF) for 10 markers of the Top10_MTAs panel for stem lodging depending on the size of the trait-customized core collection resulting from eight different sampling strategies. The underlying calculations were performed within the trait-customized core collections. Values represent mean values of 50 independent replications.

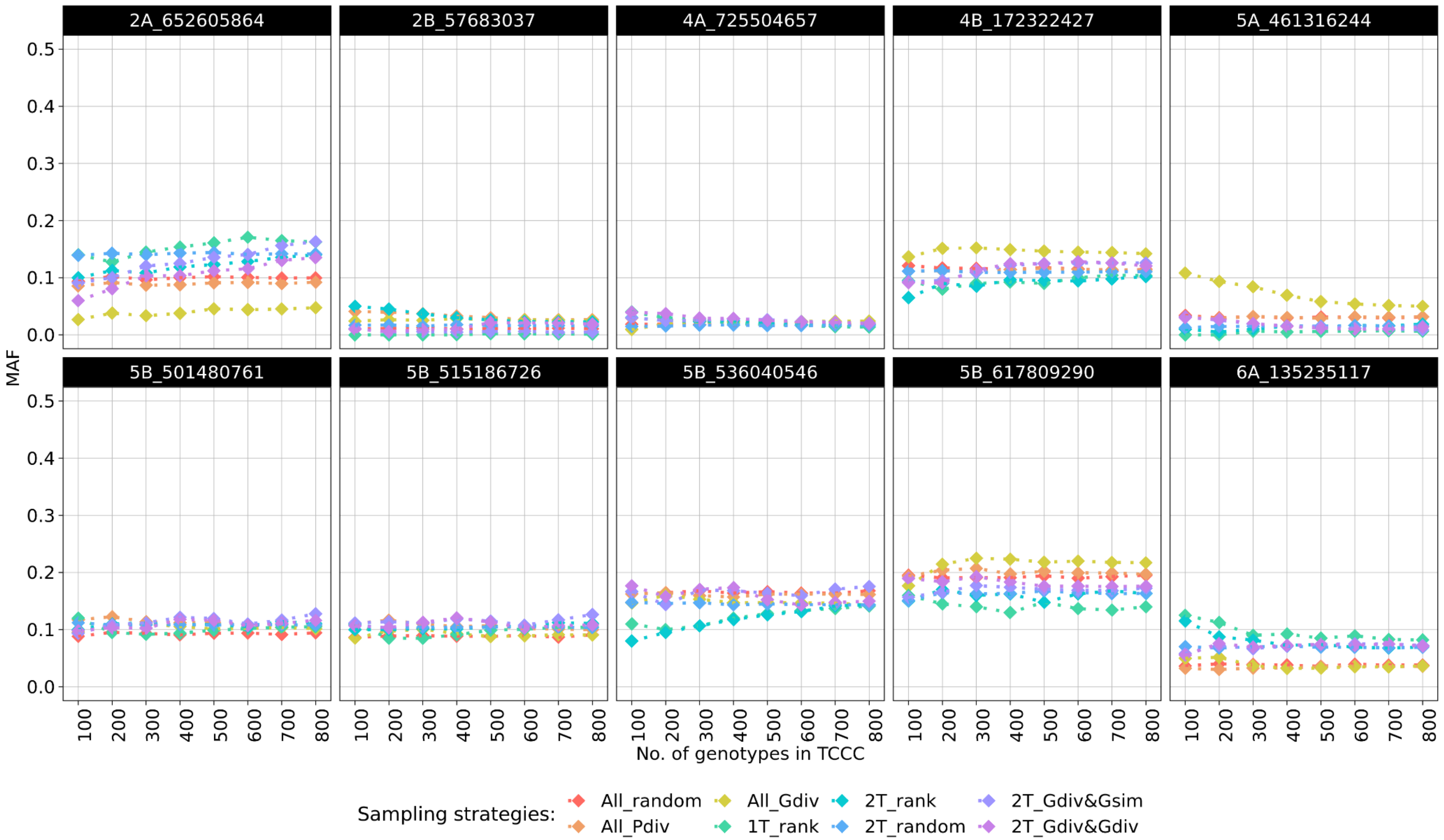

Supplementary Figure 6: Minor-allele-frequency (MAF) for 10 markers of the Top10_MTAs panel for yellow rust susceptibility depending on the size of the trait-customized core collection resulting from eight different sampling strategies. The underlying calculations were performed within the trait-customized core collections. Values represent mean values of 50 independent replications.

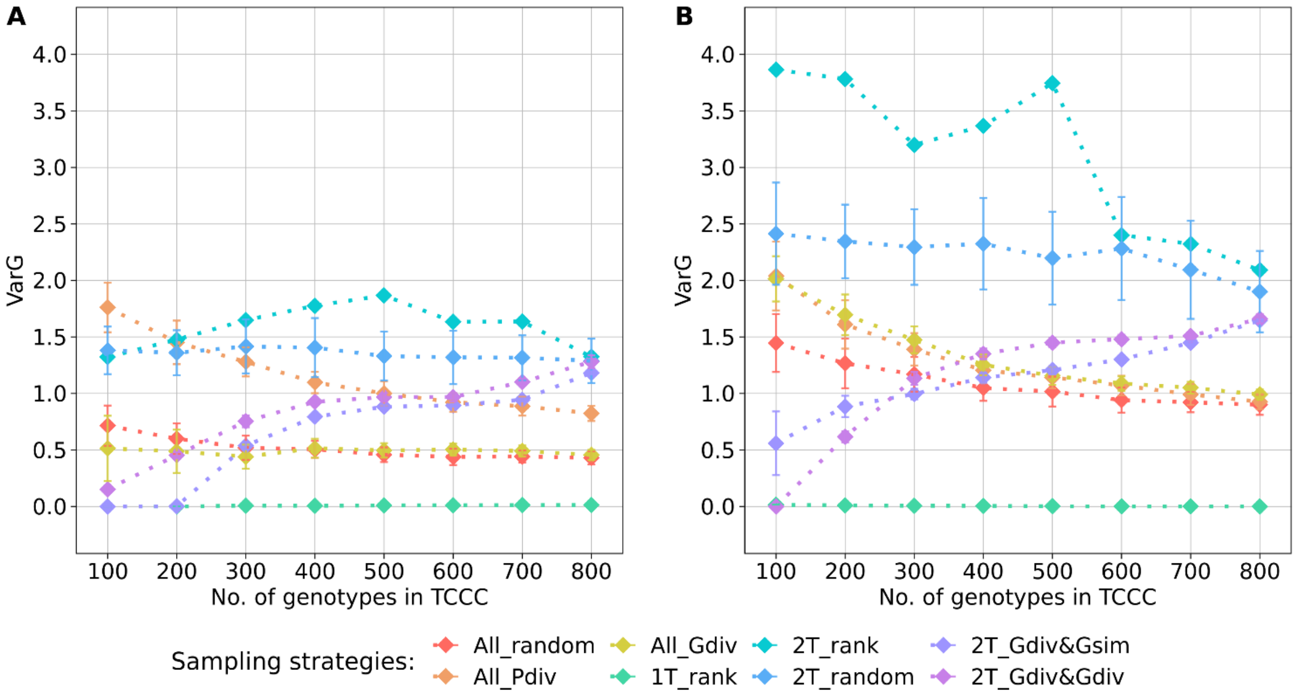

Supplementary Figure 7: Variance component for the genotype (VarG) derived from a GBLUP model depending on the number of accession samples included in trait-customized core collections for yellow rust susceptibility (A) and stem lodging (B), respectively. Values are depicted separately for eight different sampling strategies and represent means of 50 independent replications; whiskers display the standard deviations.

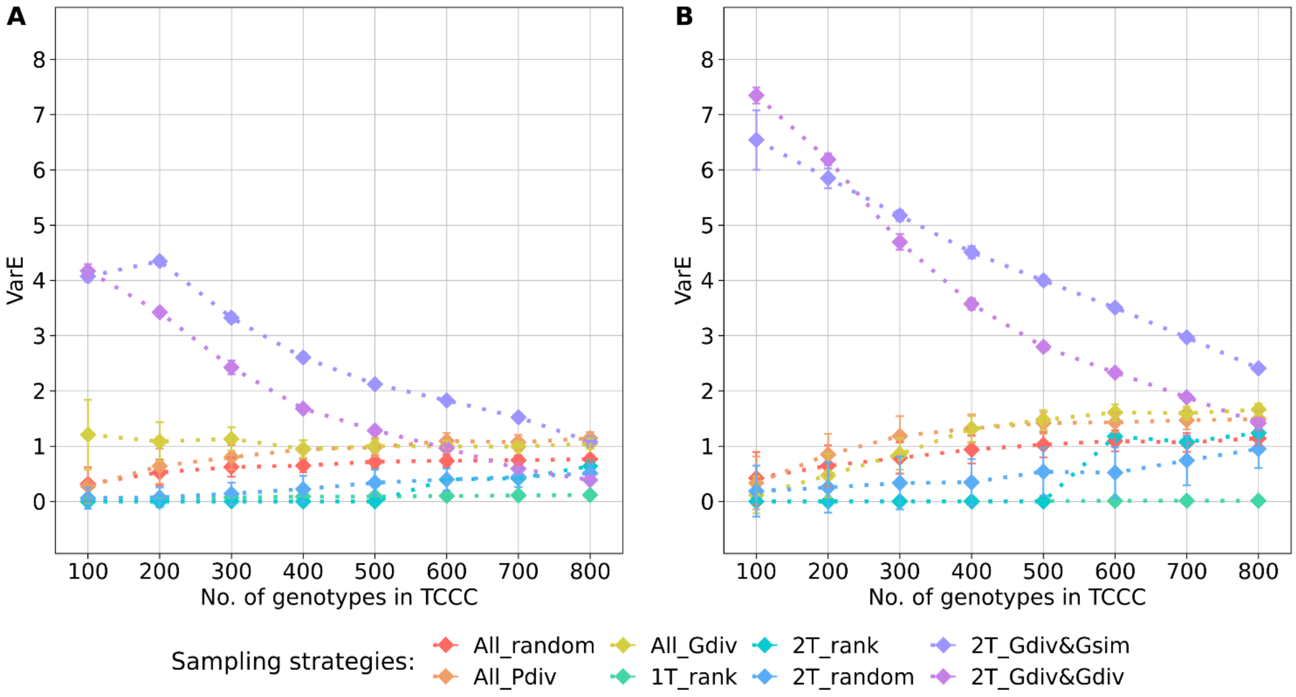

Supplementary Figure 8: Variance component for the residual term (VarE) derived from a GBLUP model depending on the number of accession samples included in trait-customized core collections for yellow rust susceptibility (A) and stem lodging (B), respectively. Values are depicted separately for eight different sampling strategies and represent means of 50 independent replications; whiskers display the standard deviations.

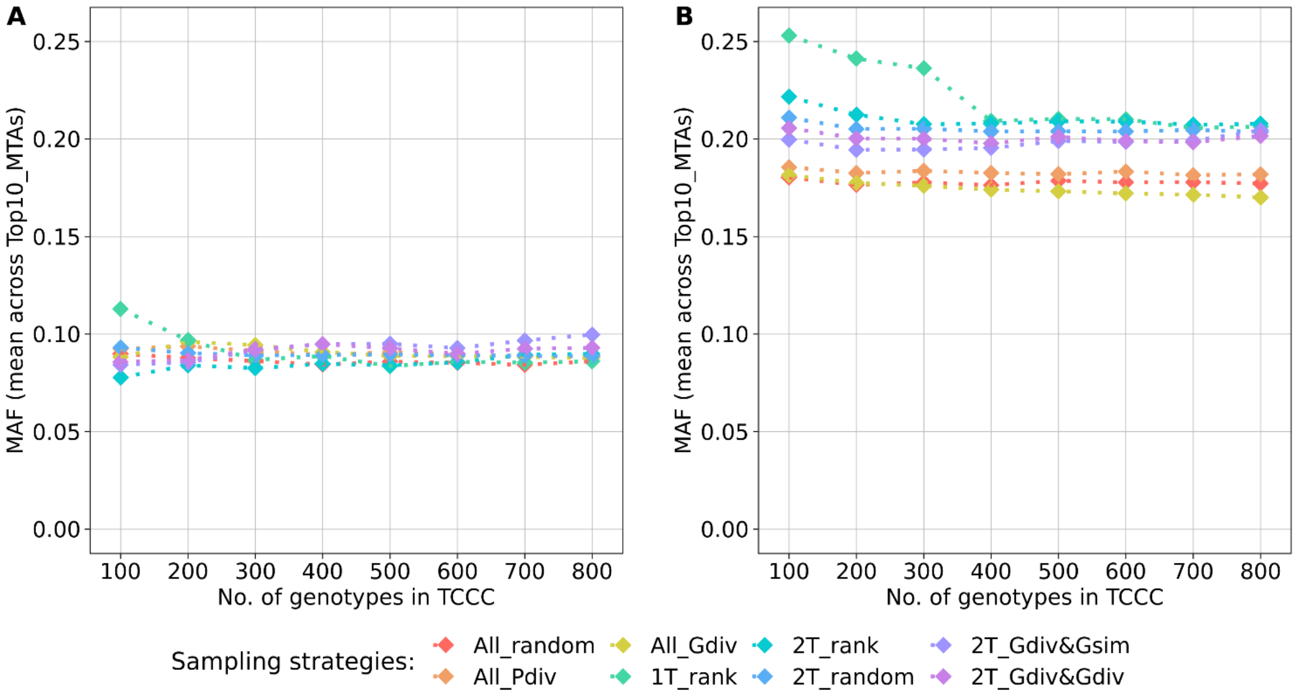

Supplementary Figure 9: Mean of minor-allele-frequency (MAF) for the 10 markers of the Top10_MTAs marker panel depending on the number of accession samples included in trait-customized core collections for yellow rust susceptibility (A) and stem lodging (B). Values are depicted separately for eight different sampling strategies and represent means of 50 independent replications; whiskers display the standard deviations.

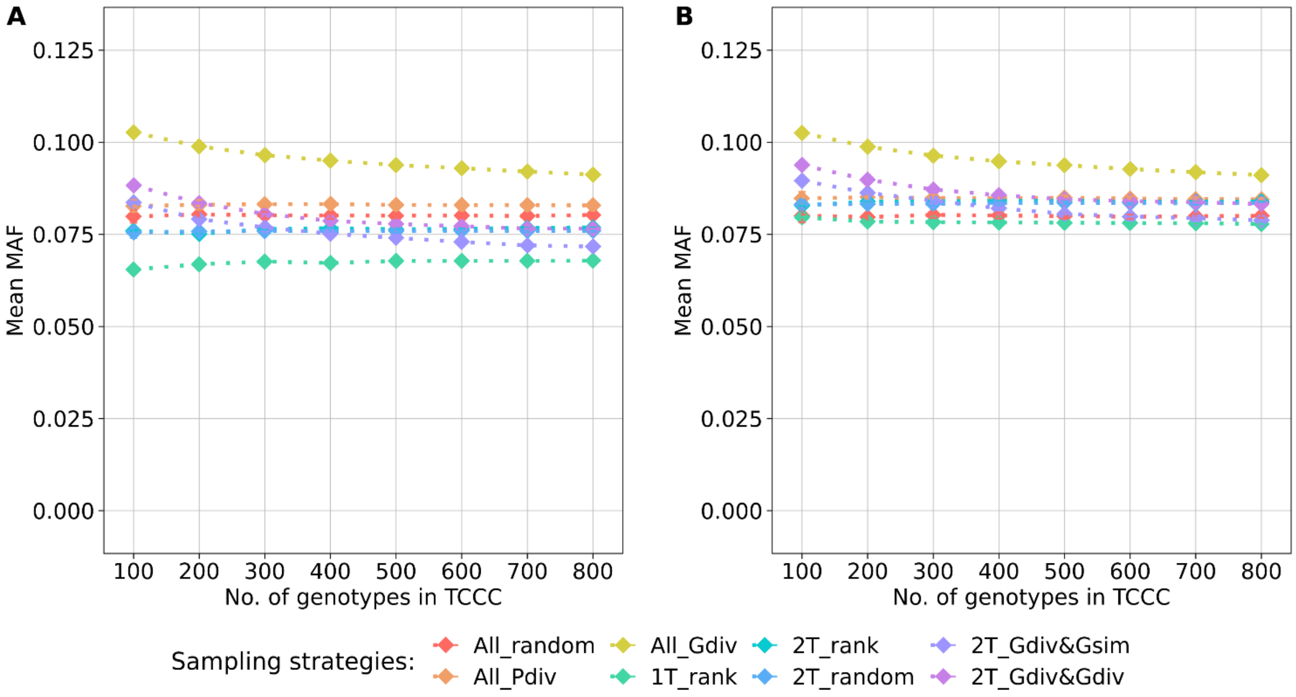

Supplementary Figure 10: Mean of minor-allele-frequency (MAF) for all markers depending on the number of accession samples included in trait-customized core collections for yellow rust susceptibility (A) and stem lodging (B). Values are depicted separately for eight different sampling strategies and represent means of 50 independent replications; whiskers display the standard deviations.
